# THz‐Driven Coherent Phonon Fingerprints of Hidden Symmetry Breaking in 2D Layered Hybrid Perovskites

**DOI:** 10.1002/adma.202502204

**Published:** 2025-10-03

**Authors:** Joanna M. Urban, Michael S. Spencer, Maximilian Frenzel, Gaëlle Trippé‐Allard, Marie Cherasse, Charlotte Berrezueta‐Palacios, Prakriti P. Joshi, Alexander P. Fellows, Olga Minakova, Eduardo B. Barros, Luca Perfetti, Stephanie Reich, Martin Wolf, Emmanuelle Deleporte, Sebastian F. Maehrlein

**Affiliations:** ^1^ Department of Physical Chemistry Fritz Haber Institute of the Max Planck Society Faradayweg 4‐6 14195 Berlin Germany; ^2^ Helmholtz‐Zentrum Dresden‐Rossendorf Institute of Radiation Physics 01328 Dresden Germany; ^3^ Technische Universität Dresden Institute of Applied Physics 01187 Dresden Germany; ^4^ Lumière, Matière et Interfaces (LuMIn) Laboratory Université Paris‐Saclay ENS Paris‐Saclay CentraleSupélec CNRS Gif‐sur‐Yvette 91190 France; ^5^ Department of Physics Freie Universität Berlin 14195 Berlin Germany; ^6^ Department of Physics Universidade Federal do Ceara Fortaleza Ceara 60455‐760 Brazil; ^7^ Institut für Festkörperphysik Technische Universität Berlin Hardenbergstraße 36 10623 Berlin Germany; ^8^ Laboratoire des Solides Irradiés CEA/DRF/lRAMIS École Polytechnique CNRS Institut Polytechnique de Paris Palaiseau F‐91128 France; ^9^ Present address: Laboratoire d'Optique Appliquée ENSTA Paris CNRS École Polytechnique Institut Polytechnique de Paris 91761 Palaiseau France

**Keywords:** 2D layered perovskites, coherent phonons, hybrid metal halide perovskites, lattice dynamics, symmetry breaking, THz‐induced Kerr effect, ultrafast material control

## Abstract

Metal‐halide perovskites (MHPs) emerged as a family of novel semiconductors with outstanding optoelectronic properties for applications in photovoltaics and light emission. Recently, they also attract interest as promising candidates for spintronics. In materials lacking inversion symmetry, spin‐orbit coupling (SOC) leads to the Rashba‐Dresselhaus effect, offering a pathway for spin current control. Therefore, inversion symmetry breaking in MHPs, which are characterized by strong SOC, has crucial implications. Yet, in complex low‐dimensional hybrid organic‐inorganic perovskites (HOIPs), the presence of and structural contributions to inversion symmetry breaking remain elusive. Here, employing intense THz fields, lattice dynamics carrying spectroscopic fingerprints of inversion symmetry breaking are coherently driven and observed in Ruddlesden‐Popper (PEA)_2_(MA)*_n_*_‐1_Pb*_n_* I_3_ *_n_*_+1_ perovskites, which are globally assigned to a centrosymmetric space group. We demonstrante coherent control by THz pulses over specific phonons, which are assigned to either purely inorganic or highly anharmonic hybridized cage‐ligand vibrations. By developing a general polarization analysis for THz‐driven phonons, linear and nonlinear driving mechanisms are pinpointed. From this, simultaneous IR‐ and Raman‐activity of inorganic cage modes below 1.5 THz is identified, indicating mode‐selective inversion symmetry breaking. By exploring the driving pathways of these coherent phonons, the groundwork is laid for simultaneous ultrafast control of optoelectronic and spintronic properties in 2D HOIPs.

## Graphical abstract

Intense THz pulses are employed to drive coherent phonons in 2D Ruddlesden‐Popper hybrid metal‐halide perovskites (HOIPs) from the (PEA)_2_(MA)*_n_*_‐1_Pb*_n_* I_3_ *_n_*_+1_ family, revealing mode‐selective spectroscopic signatures of inversion symmetry breaking in those nominally centrosymmetric materials. These findings provide a handle to decode nonlinear phonon driving mechanisms toward ultrafast control of optoelectronic functionalities in low‐dimensional HOIPs.

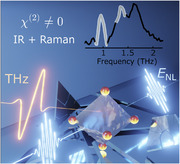

## 1 Introduction

Metal halide perovskites (MHPs) have emerged over the past decade as novel semiconductors with exceptional photophysical properties, suitable for various optoelectronic applications, including photovoltaics and light emission.^[^ ^1^ ^]^ Low‐dimensional, hybrid organic‐inorganic perovskites (HOIPs) offer enhanced stability compared to their 3D counterparts and vast structural tunability achieved via easy, solution‐based synthesis.^[^ ^2^ ^]^ Among them, layered 2D perovskites are promising candidates for light‐emitting diodes, lasers, and photodetectors.^[^ ^3^ ^]^ The remarkable properties of such low‐dimensional HOIP compounds are largely influenced by electronic confinement of the charge carriers in the inorganic sublattice^[^ ^4^ ^]^ as well as the significant coupling of the electronic and spin degrees of freedom to the vibrational excitations of the soft,^[^ ^5^ ^]^ anharmonic,^[^ ^6^ ^]^ highly polarizable^[^ ^7^ ^]^ and dynamically disordered^[^ ^8^, ^9^ ^]^ lattice. Lowered dimensionality is reported to enhance the coherent lattice dynamics in HOIPs,^[^ ^10^ ^]^ modify the phonon spectrum,^[^ ^11^, ^12^, ^13^ ^]^ and alter charge carrier‐phonon coupling,^[^ ^14^ ^]^ but the exact underlying mechanisms remain underexplored.

In addition, MHPs are recently being intensely investigated as a new promising platform for spintronic applications.^[^ ^15^, ^16^ ^]^ In conjunction with a strong spin‐orbit coupling (SOC) originating from the heavy metal atoms, the lack of inversion symmetry in MHPs leads to the emergence of the Rashba‐Dresselhaus effect:^[^ ^17^, ^18^ ^]^ a splitting of the electronic bands in momentum space, leading to an indirect bandgap and lifting of the spin degeneracy even in the absence of an external magnetic field. This makes inversion‐symmetry‐broken MHPs highly interesting for spin‐charge conversion and spin‐dependent charge transport applications. Among the inversion symmetry‐broken MHPs, materials with chiral structures allow controlling spin populations thanks to their chiroptical activity or the chiral‐induced spin selectivity (CISS) effect.^[^ ^19^ ^]^ Beyond spintronics, inversion symmetry breaking also underlies phenomena such as ferroelectricity^[^ ^20^ ^]^ and even‐order optical nonlinearities.^[^ ^21^ ^]^

Since the presence of inversion symmetry breaking in perovskite materials has far‐reaching consequences, significant research effort has recently been directed toward the chemical engineering of MHP materials with non‐centrosymmetric crystal structures to achieve new functionalities.^[^ ^22^, ^23^ ^]^ While for many perovskite materials the polar, non‐centrosymmetric crystallographic structure can be unambiguously resolved,^[^ ^24^, ^25^ ^]^ the presence and magnitude of inversion symmetry breaking in some members of the low‐dimensional MHP family remains debated. Rashba‐Dresselhaus splitting as a result of broken inversion symmetry has been reported in 2D layered Ruddlesden‐Popper perovskites,^[^ ^26^, ^27^, ^28^, ^29^ ^]^ but the exact nature of the underlying lack of inversion symmetry and conditions for the emergence of this effect remain elusive. Conflicting reports have attributed commonly studied 2D layered perovskites to either centrosymmetric or inversion symmetry‐broken static crystal structures, with the assignment often complicated by the precise determination of the orientation of organic ligands in hybrid low‐dimensional materials.^[^ ^21^ ^]^ It has been suggested that nontrivial, local inversion symmetry breaking can occur in HOIPs even in the absence of the breaking of global central symmetry,^[^ ^30^, ^31^ ^]^ leading to the emergence of the Rashba effect even in nominally centrosymmetric crystals.^[^ ^32^ ^]^ The presence and origin of such intrinsic ‘globally hidden’ symmetry breaking in HOIPs remain controversial^[^ ^33^ ^]^ and difficult to prove, especially in structurally complex, low‐dimensional hybrid compounds, highlighting the need for more detailed studies and new experimental approaches.

Especially, while most common characterization methods provide information about static material symmetries, the potential of time‐resolved methods to study the dynamic character of the symmetry breaking at ultrafast timescales has not yet been fully explored. The analysis of optical signatures of phonon modes in crystalline materials offers sensitive insights into underlying lattice symmetries, which dictate the selection rules for vibrational excitations’ interactions with light. Strong phonon‐charge carrier coupling governs many of the MHP's remarkable optoelectronic properties, including surprising defect tolerance^[^ ^34^ ^]^ and polaronic effects.^[^ ^35^ ^]^ Vibrational modes could also mediate dynamic inversion symmetry breaking and lead to the emergence of a local, dynamical Rashba effect, as previously suggested for MAPbI_3_.^[^ ^36^, ^37^ ^]^ Coherently driven phonons are therefore excellent handles for low‐energy ultrafast material control, complementary to structural and chemical engineering, as recently demonstrated in 3D^[^ ^7^ ^]^ and 2D^[^ ^10^ ^]^ MHPs.

**Figure 1:**
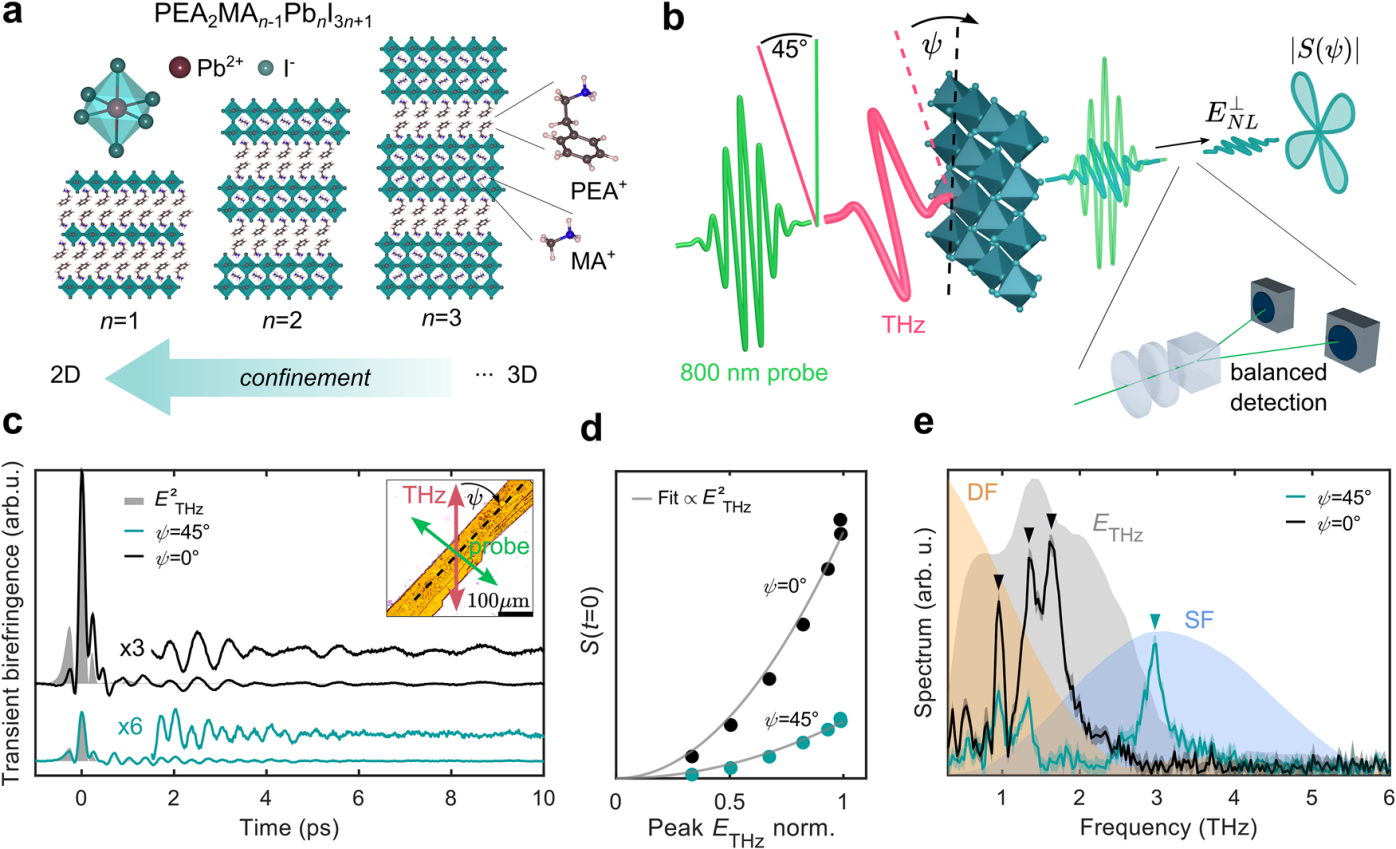
a) Schematic structure of Ruddlesden‐Popper PEA^+^‐based perovskites.^[^ ^49^ ^]^ b) Schematic of the experimental configuration. The azimuthal angle‐dependent signal *S*(ψ) is proportional to $E_{NL}^{⊥}$, the signal field component polarized perpendicular to the initial probe polarization. c) RT transient birefringence traces for the *n* = 1 sample for two different azimuthal crystal orientations. The instantaneous response approximately follows the square of the THz electric field measured by electro‐optic sampling (shaded, grey). The enlarged traces show the oscillatory signal contribution. Inset: optical microscope image of the sample with the dashed line marking the long flake edge. d) Instantaneous response peak amplitude as a function of THz electric field for different sample orientations and corresponding quadratic fits. e) FTs of the oscillatory signal in panel c with dominant peaks marked, spectrum of the THz field (shaded grey), and the spectral profile of the difference‐ (orange) and sum‐ frequency (blue) nonlinear driving force.

In this work, we therefore use intense THz fields to drive coherent vibrational modes in a family of layered 2D HOIP compounds with varying degrees of out‐of‐plane confinement (see **Figure** **1**a). The increased structural ordering, enhanced by large aromatic PEA^+^ ligands, and the high quality of our phase‐pure single crystal samples enable us to drive room temperature coherent lattice dynamics in Ruddlesden‐Popper perovskite (RPP) compounds with *n* = 1, 2, up to 3 inorganic metal‐halide octahedral layers. This contrasts with a previous study on a 2D perovskite system with short alkyl chain butylammonium ligands,^[^ ^10^ ^]^ where clear coherent dynamics was only observed in the *n* = 1 compound, highlighting the crucial role of specific ligand molecules and the interplay of the organic and inorganic sublattice, dictated by hydrogen bonding, steric effects, and electrostatic interactions.^[^ ^38^ ^]^ Through a systematic study of THz‐induced transient birefringence, we trace the evolution of lattice dynamics as a function of the number of inorganic layers *n*, identifying a vibrational mode likely specific to the organic‐inorganic sublattice interface. Our comprehensive analysis of mode symmetries and driving mechanisms identifies a globally hidden inversion symmetry breaking, evidenced by the simultaneous Raman and IR activity^[^ ^39^ ^]^ of specific inorganic layer phonons. We demonstrate how tailoring excitation polarization conditions allows the selective targeting of specific vibrational modes, leveraging symmetry properties for ultrafast lattice‐based material control.

## 2 Results

Here, we employ intense single‐cycle THz pump pulses with peak fields of ≈1 MV cm^−1^ (see Experimental Section) and collinearly propagating 800 nm probe pulses, linearly polarized at 45° with respect to the THz, to stroboscopically sample the dynamics of the THz‐induced birefringence^[^ ^7^, ^40^ ^]^ (see Figure 1b). The signal field is emitted in a wave mixing process by the nonlinear polarization induced via THz and visible/NIR field interactions.^[^ ^7^ ^]^ The polarization component of the signal field perpendicular to the probe field is detected in a balanced detection scheme (see Figure 1b; Section S17 and S18, Supporting Information). The anisotropic signal generation in this nonlinear process can effectively be described as a rotation of the probe polarization by the angle ϕ due to transient changes of the refractive index (see Figure 1b). The probe photon energy is below the band gap of all the investigated samples, allowing us to investigate lattice dynamics in the unperturbed electronic ground state. With this method, we investigate single‐crystal samples of lead iodide compounds from the family of layered Ruddlesden‐Popper perovskites (RPPs) (PEA)_2_MA*_n_*_‐1_Pb*_n_* I_3_ *_n_*_+1_ (see Figure 1a; Experimental Section, and Figure S1, Supporting Information). In hybrid RPPs, large organic cations (also called ligands), in our case phenethylammonium [C_6_H_5_NH_3_]^+^ (PEA^+^), form bilayers separated by a van der Waals gap, which act as spacers between the slabs built from *n* planes of inorganic metal‐halide octahedra.^[^ ^41^ ^]^ Additionally, smaller cations, in our case, methylammonium [CH_3_NH_3_]^+^ (MA^+^), are incorporated in the 12‐fold coordination in between the PbI octahedra in the inorganic sublattice for *n ≥* 2. The ligands interact with each other and with the octahedral lattice via multiple non‐covalent bonds, which enable vibrational coupling between the organic and inorganic lattice.^[^ ^42^ ^]^

First, we measure the THz pump‐induced transient birefringence for the *n* = 1 sample at room temperature, varying the azimuthal crystal orientation in the plane perpendicular to the natural cleavage plane (ψ = 0° azimuthal angle is defined as the long macroscopic crystal edge being parallel to the THz polarization, see inset Figure 1c). Both beams are incident normal to the sample surface, which corresponds to the 2D perovskite layer plane. Figure 1c shows the transient birefringence signal in (PEA)_2_PbI_4_ consisting of an instantaneous electronic response, which follows the square of the THz electric field, and a longer‐lived oscillatory component. The THz‐induced Kerr effect^[^ ^40^ ^]^ (TKE) nature of the instantaneous signal is confirmed by the quadratic dependence of the instantaneous response peak at pump‐probe delay *t* = 0 on the THz electric field (see Figure 1d). Furthermore, its amplitude shows a clear fourfold anisotropy (Figure S7, Supporting Information), consistent with the fourfold pattern observed for visible‐range nonlinear susceptibility in (PEA)_2_PbI_4_.^[^ ^43^ ^]^ Based on this strong instantaneous nonlinear response, a nonlinear THz refractive index can be estimated as *n*_2_ ≈ 1.4  × 10^−12^ cm^2^ W^−1^ (lower limit, see Section S5, Supporting Information). This value is higher than for the related 3D compound MAPbBr_3_ and several orders of magnitude higher than for many typical materials for nonlinear THz photonics.^[^ ^7^ ^]^ This finding complements previous reports of very high optical nonlinearities of 2D perovskites in the visible frequency range.^[^ ^44^, ^45^ ^]^

We assign the oscillatory part of the signal at longer times to coherent Raman‐active phonon signatures, which decay over the course of several picoseconds.^[^ ^10^ ^]^ The Fourier transform (FT) of this oscillatory signal (calculated for a cut‐off time of *t* > 1.5 ps to exclude contributions from the instantaneous electronic response, Figure 1e) reveals multiple peaks in the 0.5–3 THz range, within the spectral bandwidth of the pump THz field and its nonlinear sum‐ and difference‐frequency driving forces.^[^ ^46^ ^]^ This strongly contrasts with the single low‐frequency mode dominating the transient birefringence response of 3D MAPbBr_3_,^[^ ^7^ ^]^ which only becomes apparent at cryogenic temperatures due to strong phonon damping. Notably, a previous TKE study on 2D perovskites with butylammonium ligands revealed only a single vibrational mode at ≈1.8 THz.^[^ ^10^ ^]^ The larger number of Raman‐active modes observed by us is likely due to the lower, triclinic crystal symmetry of (PEA)_2_PbI_4_ ($\bar{P1}$ space group^[^ ^32^, ^47^ ^]^). The long‐lived room temperature vibrational lattice coherence in our investigated 2D compound, significantly enhanced compared to the 3D MHPs, enables systematic studies of the phonon‐modulated anisotropic transient birefringence response as a function of the sample azimuthal angle ψ. The coherence decay times of the most prominent modes, marked by the triangles in Figure 1e), are 3.5, 1.5, and 2 ps for the 0.9, 1.7, and 2.9 THz modes, respectively. These timescales agree with the estimated 2.4 ps decay time of the single 1.8 THz phonon mode observed in (BA)_2_PbI_4_ (Ref. [10]). Comparing the coherent phonon signals from several crystals of significantly different thicknesses allowed us to carefully exclude propagation effects as the origin of the long‐lived oscillatory signals^[^ ^7^, ^48^ ^]^ (see Figure S2, Supporting Information) and confirm their assignment as vibrational lattice modes.

Based on previous theoretical and Raman spectroscopy studies, we assign the low‐frequency 0.9, 1.4, and 1.7 THz modes predominantly to vibrations of the PbI cage.^[^ ^50^, ^51^ ^]^ In 2D HOIPs, modes in this frequency range have been previously identified as tilts and rotations of the octahedral cages^[^ ^50^, ^51^, ^52^ ^]^ (≈0.8 THz), predominantly in‐plane^[^ ^50^ ^]^ metal‐halide bond bending and stretching (1.3–1.7 THz)^[^ ^50^, ^52^, ^53^, ^54^ ^]^ as well as rattling and torsional modes of both the large ligand molecules and the smaller organic cations inside the inorganic cage (0.8 and 1.7 THz).^[^ ^54^, ^55^ ^]^ The 1.7 THz mode is close in frequency to the single dominating 1.8 THz Raman‐active phonon in (BA)_2_PbBr_4_ assigned to the inorganic cage.^[^ ^10^ ^]^ The relative shift in frequency between the two compounds is attributed to the larger mass of the iodide compared to the bromide ions. Driving these low‐frequency metal halide cage modes is key to ultrafast control of RPP optoelectronic properties, as they are known to strongly modulate the electronic structure of (PEA)_2_PbI_4_ and related compounds.^[^ ^56^, ^57^ ^]^ The mode ≈2.9 THz in 2D HOIPs has previously been assigned to either out‐of‐plane metal‐halide stretching^[^ ^52^ ^]^ or to a hybridized mode with vibrational contributions from both the inorganic sublattice and the organic ligand cation.^[^ ^53^ ^]^ A very short‐lived 2.9 THz vibration was reported in MAPbI_3_ by a recent THz‐driven broadband optical spectroscopy study^[^ ^58^ ^]^; however, it appeared at extremely short times (*t* < 1.5 ps) during the THz excitation, for which the coherent phonon signal cannot be reliably extracted in the case of our experimental method.^[^ ^7^ ^]^ We did not observe this mode in structurally similar 3D MAPbBr_3_,^[^ ^7^ ^]^ nor in multiple A‐site cation 3D bromide perovskites^[^ ^59^ ^]^ in TKE experiments in the 80–295 K range, meaning that the 2.9 THz vibration is either completely absent, or too strongly damped to be observed. The prominence and long coherence time of this phonon thus appear to be characteristic to our 2D system. Given the evidence of enhanced lattice coherence times in the *n* = 1 PEA^+^‐based RPPs compared to 3D HOIP compounds and the appearance of multiple previously unreported vibrational modes, we next investigate the dependence of the lattice dynamics on the degree of confinement by comparison with *n* = 2, 3 samples.

**Figure 2:**
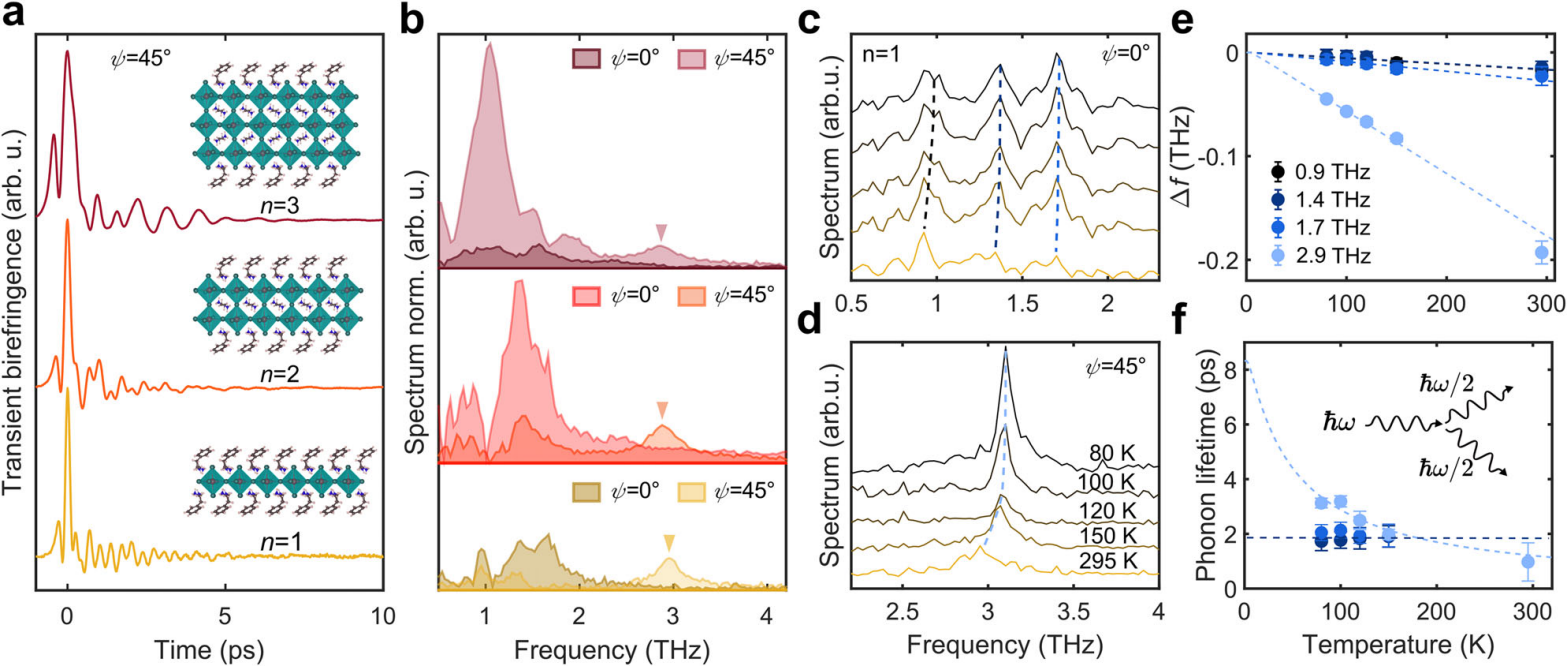
a) RT transient birefringence traces for RPPs *n* = 1, 2, 3, normalized to the instantaneous peak amplitude. b) FTs of the oscillatory signal for the RPPs at ψ = 45° and ψ = 0° crystal edge orientation with the 2.9 THz mode marked. c) Temperature dependence of the spectra for *n* = 1 measured at ψ = 0° showing the low‐frequency cage modes. The dashed lines mark the evolution of the characteristic peaks. d) Temperature dependence of the spectra for *n* = 1 measured at ψ = 45°, showing the evolution of the 2.9 THz mode. e) Relative frequency shift of the phonon modes with temperature and predictions of a 3‐phonon scattering model for the different modes (dashed lines). f) Lifetimes of the modes as a function of temperature, estimated by a 3‐phonon scattering model for the 2.9 THz mode (light blue dashed line) and approximately temperature‐independent lowest‐frequency mode lifetime (dark blue dashed line). Inset: schematic of symmetric 3‐phonon scattering.

Transient birefringence measurements at room temperature are compared in **Figure** **2**a for *n =* 1, 2, and 3 for crystal edge orientation at 45° to the THz polarization direction (see also Figure S1, Supporting Information). Here, we witness clear coherent phonon signatures for *n* = 2, 3, in contrast to previously investigated butylammonium/methylammonium (BA/MA)‐based RPP compounds, which only displayed coherent dynamics for *n* = 1.^[^ ^10^ ^]^ We attribute this difference to a higher structural ordering imposed by the bulky, rigid PEA^+^ cations, which can undergo π − π stacking.^[^ ^60^ ^]^ Figure 2b shows the corresponding FT amplitude spectra of the oscillatory signals (for *t* > 1.5 ps), for two crystal orientations ψ = 0° and ψ = 45°. Coherent phonon dynamics in pure PEA^+^ ligand‐halide salt crystals (see Section S15, Supporting Information) significantly differ from those in the perovskite crystals, confirming the observed lattice dynamics in RPPs as an emergent property of the entire organic‐inorganic structure.^[^ ^54^ ^]^

As shown in Figure 2b, we observe a clear amplitude decrease of the 2.9 THz mode relative to the low‐frequency cage modes with an increasing number of inorganic layers from *n* = 1 to *n* = 3. We therefore tentatively assign the 2.9 THz to altered vibrational dynamics at the interface between the organic spacer ligands and the inorganic cage layers. The decrease in the mode's relative amplitude with increasing number of layers *n* would, in this case, arise from a reduced dominance of the organic‐inorganic interface. The mode could be related to rigid‐body dynamics of the PEA^+^ cation^[^ ^53^, ^54^ ^]^ or to inorganic cage vibrations activated in the altered local environment of the outermost inorganic octahedral layer interfacing the ligands.^[^ ^54^ ^]^ Recently reported ab initio calculations predict higher mobility and mean displacements of halogen ions at the interface, leading to significantly larger vibrational density of states above 2.5 THz compared to the intralayer ions, supporting this interpretation.^[^ ^61^ ^]^ Alternatively, it could be a purely inorganic cage mode, more strongly damped in the higher‐*n* and 3D compounds due to the presence of MA^+^ cations.^[^ ^10^, ^62^ ^]^

To gain further insight into the vibrational modes’ nature, we characterize the lattice dynamics for the *n* = 1 sample as a function of temperature. Figure 2c,d show the FTs of the transient birefringence signals in the temperature range from 80 to 295 K. The lower‐frequency modes (Figure 2c, ψ = 0°) and the 2.9 THz mode (Figure 2d, ψ = 45°) slightly soften and broaden with increasing temperature. However, no significant spectral changes are observed. This confirms the absence of phase transitions in this temperature range and is consistent with previous Raman spectroscopy studies.^[^ ^47^ ^]^ The softening of vibrational modes with temperature is a result of lattice anharmonicity,^[^ ^63^ ^]^ which underlies both lattice expansion and phonon–phonon scattering (see Section S10, Supporting Information). We plot the relative shift of the four characteristic modes in Figure 2e along with predictions of a simple model considering only a symmetric anharmonic decay into two lower‐frequency phonons^[^ ^64^ ^]^ (inset of Figure 2f, see Section S10, Supporting Information). Strikingly, the 2.9 THz mode shifts significantly more with temperature than all the low‐frequency modes, suggesting the presence of more efficient anharmonic decay channels. The temperature dependence of the extracted phonon lifetimes (see Figure 2f) is compared to a model including defect scattering^[^ ^65^ ^]^ and temperature‐dependent phonon–phonon scattering^[^ ^63^ ^]^ (see Section S10, Supporting Information). This simple model qualitatively captures the observed evolution and highlights the much higher anharmonicity of the 2.9 THz mode compared to the lower frequency modes. At low temperatures, the 2.9 THz mode has a longer coherence time than the inorganic cage modes (τ_1.4THz_ ≈ 1.7 ps,   τ_1.7THz_ ≈ 2 ps,  τ_2.9THz_ ≈ 3 ps at 80 K). At room temperature, the situation is reversed and the 2.9 THz mode is more strongly damped (τ_2.9THz_ ≈ 1 ps) while the inorganic mode lifetimes are only very slightly affected by temperature (as can also be seen comparing the relative linewidths of the 0.9 and 2.9 THz modes at RT in Figure 1e). In line with our tentative assignment of this mode to a hybridized ligand‐inorganic vibration, we propose that the strong decrease of its lifetime with temperature is due to scattering with thermally activated vibrations at the organic‐inorganic interface. Despite the lack of direct bonds between the organic and inorganic layers in RPPs, significant vibrational coupling between the organic and inorganic sublattice vibrations can occur due to mechanical and electrical anharmonicities.^[^ ^42^ ^]^ Strong anharmonicity in RPPs is known to lead to fast phonon dephasing, compared to, for example, 2D lead organic chalcogenides with direct inorganic‐ligand connectivity and a more rigid crystal framework.^[^ ^66^ ^]^ The stability of hydrogen bonds between the ligand molecules and halide ions in HOIPs strongly decreases with temperature,^[^ ^67^ ^]^ potentially increasing dynamic disorder and reducing the lattice coherence. Weakening of the non‐covalent interactions mediating the organic‐inorganic sublattice coupling may explain the softening of a hybridized organic‐inorganic mode at higher temperatures. The different scattering rates for the specific vibrations may also reflect distinct selection rules for the scattering processes imposed by local symmetries of the respective lattice subsystems.^[^ ^66^ ^]^ The observed nontrivial evolution of phonon amplitudes and lifetimes is a signature of strong anharmonicity and nonlinear phonon–phonon coupling in hybrid 2D layered perovskites.

**Figure 3:**
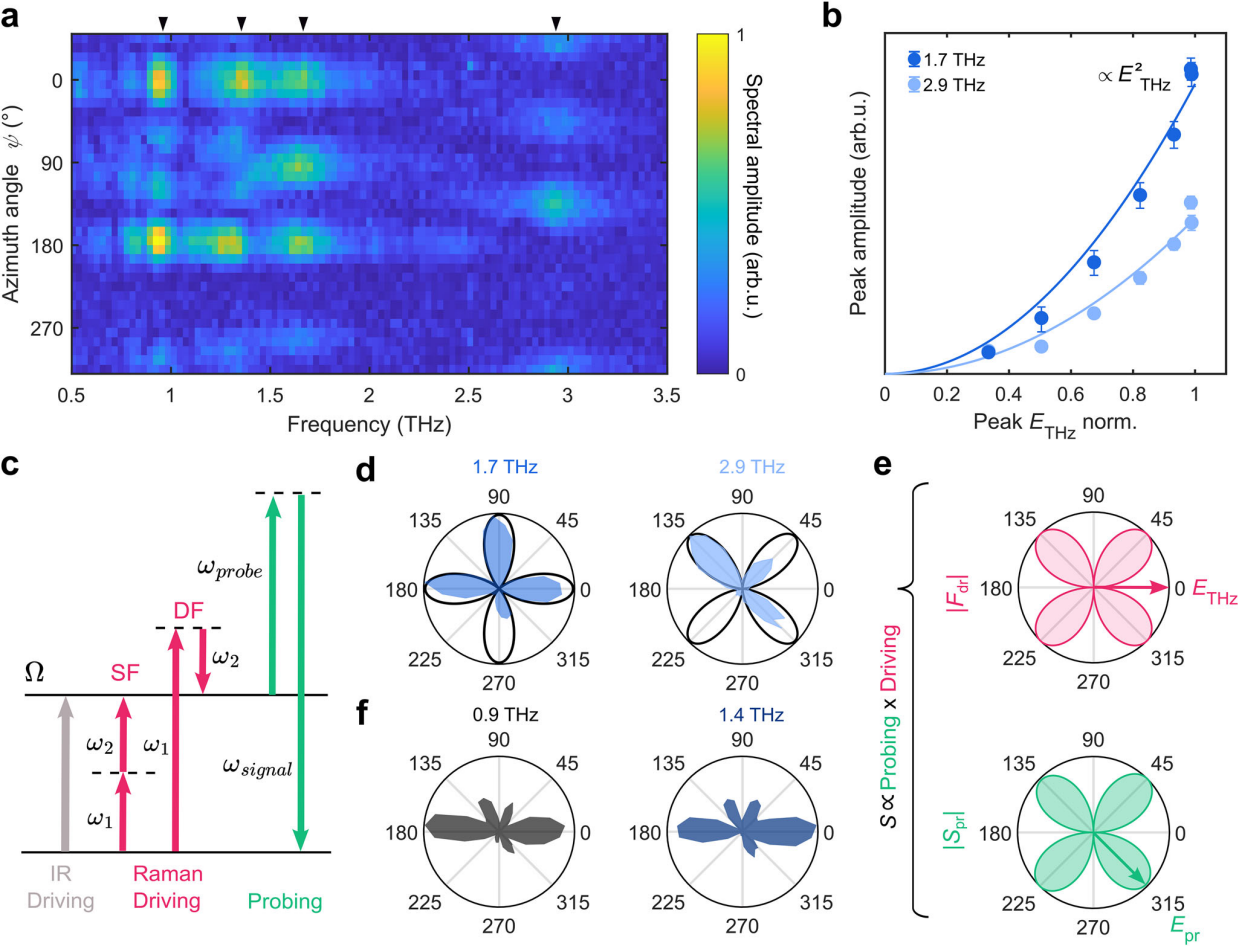
a) Azimuthal angle dependence of the spectral amplitude of the transient birefringence FT for the *n* = 1 sample. ψ = 0° corresponds to the crystal long axis oriented parallel to the THz electric field. b) Amplitude of the 1.7 and 2.9 THz modes as a function of the peak THz electric field and quadratic fits c) Schematic of nonlinear difference‐ and sum‐frequency Raman excitation driving by THz fields with frequencies ω_1_ and ω_2_ (magenta arrows) and Raman‐type anti‐Stokes probing mechanism (green arrows). For comparison, linear (IR) driving is shown by a gray arrow. d) Amplitude of the 1.7 and 2.9 THz FT peaks as a function of the sample's azimuthal angle (shaded area) and theoretically calculated TKE signal amplitudes (black solid line). e) Amplitude of Raman‐type driving force |*F*_dr_| and detection efficiency |*S*_pr_| as a function of sample azimuthal rotation angle. Arrows indicate the polarization directions of the THz and incident probe electric fields. f) Amplitude of the 0.9 and 1.4 THz modes as a function of sample azimuthal angle.

To further investigate the intriguing anisotropy of the transient birefringence signal and the driving mechanisms of the coherent lattice modes, we perform measurements as a function of crystal orientation relative to the pump and probe polarization directions. In this way, we can systematically analyze the symmetries of the phonon modes. Here, we focus on the results and analysis for the simplest case of the *n* = 1 sample, while the corresponding results for *n* = 2,3 are shown in Section S13 (Supporting Information). In **Figure** **3**a we map the measured spectral amplitude as a function of rotation angle ψ in the inorganic layer plane. The four dominant modes ≈0.9, 1.4, 1.7, and 2.9 THz, exhibit distinct angular signatures. Figure 3b shows the clear quadratic dependence of the 1.7 and 2.9 THz mode amplitude on the THz electric field. This scaling suggests a nonlinear driving mechanism, which could be either Raman‐type photonic excitation,^[^ ^46^, ^68^ ^]^ where two THz photons within the bandwidth of the broadband THz pulse excite a Raman‐active vibration Ω_*R*_ in a sum‐frequency^[^ ^46^ ^]^ or difference‐frequency process (as shown in Figure 3c), or ionic Raman‐type excitation, via the anharmonic coupling of two directly driven IR‐active phonons.^[^ ^68^ ^]^ The direct (i.e., linear) excitation pathway (also shown for comparison in Figure 3c) remains forbidden for a Raman‐active mode in the absence of inversion symmetry breaking, due to the rule of mutual exclusion, which prohibits simultaneous IR‐ and Raman‐activity.^[^ ^69^ ^]^

In the following, we show how the polar patterns observed for the dominant modes (shown in Figure 3d,e) can be explained in the framework of the nonlinear photonic driving for the 1.7 and 2.9 THz phonons, and how they allow us to unveil the specific mode symmetries. To do this, we separately consider the Raman‐type coherent phonon driving and probing processes (see Figure 3c). The measured signal amplitude *S* is proportional to the product of the driving force $F_{\mathrm{dr}}^{R}$ and probing sensitivity *S*_pr_, i.e. $S\propto S_{\mathrm{pr}}F_{\mathrm{dr}}^{R}$. In the two‐photon excitation scheme, the coherent phonon at frequency Ω_R_ = ω_THz,1_ ± ω_THz,2_ is driven by the interaction of the system with two THz fields *E* ^THz,1^ and *E* ^THz,2^ at frequencies ω_THz,1_ and ω_THz,2_, which perturb the electronic cloud and create a driving force $F_{\mathrm{dr}}^{R}$ along the vibrational mode coordinate. The driving force is proportional to (see details in Section S17, Supporting Information):

(1) $$\begin{array}{c} F_{\mathrm{dr}}^{R}\propto E^{\mathrm{THz},2}\cdot R^{\omega_{\mathrm{THz},1},\Omega_{R}}E^{\mathrm{THz},1} \end{array}$$

where $R^{\omega_{\mathrm{THz},1},\Omega_{R}}$ is the Raman susceptibility tensor. The values of the tensor components are dictated by the Ω_R_ mode symmetry and generally depend on the driving field frequencies ω_THz,1_ and ω_THz,2_. The probing is also described as a Raman‐type process (as shown in Figure 3c), in which the probe field *E* ^pr^ is scattered by the coherent lattice vibrations, which give rise to Stokes and anti‐Stokes sidebands, which simultaneously contribute to the measured signal within the spectral bandwidth of the fs probing pulse. The scattered field components polarized perpendicular to the probe field's polarization are detected and give rise to the anisotropic signal (see Section S17, Supporting Information for full description). For simplicity, only the more intuitive anti‐Stokes probing pathway is shown schematically in Figure 3c. The Stokes pathway also contributes to the measured signal, but carries equivalent information, as shown in the full WMEL and Feynman diagrams (in Figure S25, Supporting Information). The probing sensitivity *S*_pr_ is proportional to $S_{\mathrm{pr}}\propto {\hat{e}}_{\mathrm{pr}}^{⊥}\cdot R^{\omega_{\mathrm{pr}},\Omega_{R}}\;E^{\mathrm{pr}}$, where $R^{\omega_{\mathrm{pr}},\Omega_{R}}$ is the Raman tensor of the mode, defined for the incident probe field frequency ω_pr_ (see Supporting Information), and ${\hat{e}}_{\mathrm{pr}}^{⊥}$ a unitary vector perpendicular to the probe polarization.

The Raman‐active modes in crystals belonging to the triclinic $\bar{P1}$ space group has *A_g_* symmetry. In principle, all Raman tensor elements for such modes can be nonzero.^[^ ^70^ ^]^ We note, however, that only the anisotropic contributions to the Raman tensor elements give rise to the scattered field components which are detected in our balanced detection scheme. We find that the polar dependence of the TKE signal for the 1.7 and 2.9 THz modes can be very well reproduced based on tensors obtained by fitting the polar dependence of their intensity in polarization‐resolved spontaneous Raman scattering experiments (see Section S14, Supporting Information) by:

(2) $$\begin{array}{c} R^{\alpha }=\left[\begin{array}{cc} a & \frac{4}{3}ae^{i\pi /2}\\ \frac{4}{3}ae^{i\pi /2} & -a \end{array}\right],\;R^{\beta }=\left[\begin{array}{cc} be^{i\pi /2} & -\frac{8}{9}b\\ -\frac{8}{9}b & -be^{i\pi /2} \end{array}\right] \end{array}$$

To describe the probing at 800 nm we assume tensors $R^{\omega_{\mathrm{pr}},\Omega_{R}}$ identical to the ones obtained from fitting the spontaneous Raman scattering data, and use the real part of these tensors as $\;R^{\omega_{\mathrm{THz},1},\Omega_{R}}$ to construct the driving force $F_{\mathrm{dr}}^{R}\propto E^{\mathrm{THz},2}\cdot R^{\omega_{\mathrm{THz},1},\Omega_{R}}E^{\mathrm{THz},1}$. We calculate the predicted azimuthal angle dependence for the 1.7 THz and 2.9 THz mode amplitudes, using **R** ^α^ and **R** ^β^ and their real parts respectively, as shown with black lines in Figure 3d. The deviations from the calculated pattern are predominantly artifacts due to inhomogeneities of the rotating sample (see also Figure S7, Supporting Information). We summarize this mechanistic concept for the 2.9 THz mode in Figure 3e, which shows the calculated azimuthal angle dependence of the driving force $|F_{\mathrm{dr}}^{R}|$ and the probing sensitivity |*S*_pr_| amplitudes based on $R^{\beta }$. Their product matches very well the experimentally extracted 2.9 THz mode amplitude variation. Performing the same calculation using a tensor of the form $R^{\alpha }$ allow us to reproduce the 1.7 THz mode's behavior.

**Figure 4:**
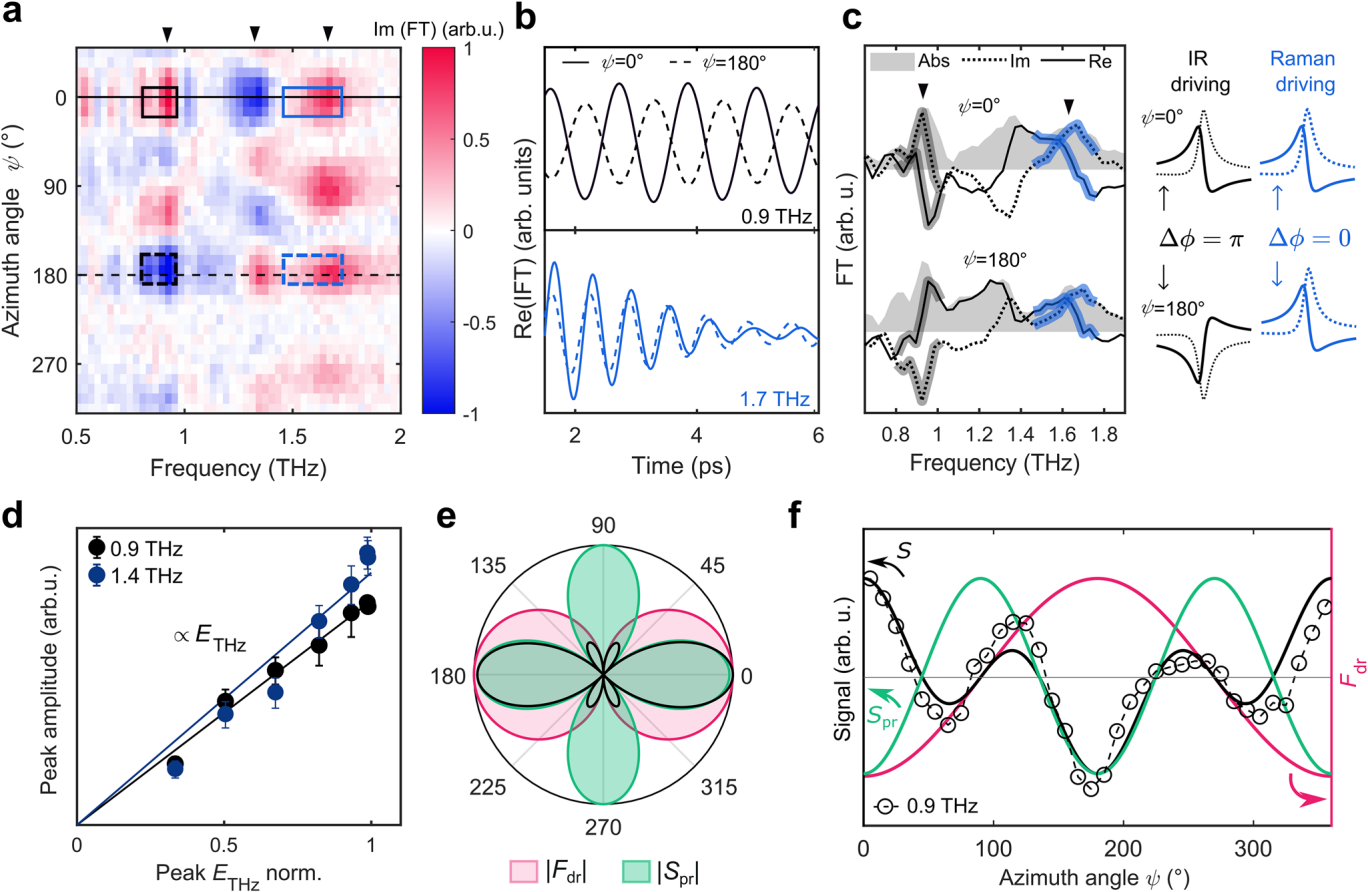
a) Normalized imaginary part of the FT of the transient birefringence signal as a function of the sample azimuthal angle for the *n* = 1 sample. The boxes marked with solid (dashed) lines indicate regions in which the signal was averaged around ψ = 0° (ψ = 180°) for calculating the inverse FT. b) Inverse FTs of the masked regions in panel a), showing the time domain signal corresponding to the 0.9 and 1.7 THz phonon modes at ψ = 0° (solid) and ψ = 180° (dashed). c) The amplitude (shaded gray area) and real (solid) and imaginary (dotted) parts of the FT around ψ = 0° and ψ = 180° with highlighted spectral regions used to obtain traces shown in (b). Sketches on the right show the behavior of the complex FT upon 180° sample rotation for an IR‐ and Raman‐driven resonance. d) Amplitude of the 0.9 THz and 1.4 THz modes as a function of the THz field and linear fits. e) Theoretically calculated amplitude of the driving force (shaded magenta), probing sensitivity (green) and detected transient birefringence signal (black) as a function of the azimuthal angle f) Azimuthal angle dependence of the 0.9 THz mode amplitude, multiplied by the sign of the FT imaginary part to illustrate the changes of the phase (circles), the theoretically calculated transient birefringence signal *S* (black), driving force $F_{\mathrm{dr}}^{\mathrm{IR}}$ (magenta) and probing sensitivity *S*_pr_ (green).

In striking contrast, the 0.9 and 1.4 THz modes show a six‐lobed polar amplitude pattern (see Figure 3f), which cannot be modeled well assuming a Raman‐type driving and detection mechanism (see Section S12, Supporting Information). In the following, we show that this experimental result can be explained by assuming simultaneous IR and Raman activity of these modes, which is a clear sign of broken inversion symmetry.^[^ ^69^ ^]^ To decode the peculiar signal symmetry, we make use of the phase information provided by the coherent nature of our time‐domain method and analyze the full complex FT. **Figure** **4**a shows the imaginary part of the FT of the signal as a function of the azimuthal angle ψ to illustrate the phase changes of the oscillatory phonon signal with sample rotation. A sample rotation by 180° is equivalent to inverting the THz field polarity ($E^{\mathrm{THz}}\to -E^{\mathrm{THz}}$) relative to the crystal. Comparing the inverse FTs of the frequency filtered 0.9 and 1.7 THz modes (see box masks in Figure 4a) in Figure 4b we observe a clear π phase shift of the oscillatory signal for the 0.9 THz mode and an unchanged phase of the 1.7 THz mode upon 180° rotation. The π phase shift corresponds to a change of sign of both the real and imaginary part of the complex FT, as seen in the phase map of Figure 4a and demonstrated in Figure 4c.

To rationalize this observation, we assume a linear driving via the coupling of the THz field to the electric dipole moments of IR‐active 0.9 and 1.4 THz modes. In contrast to the Raman‐type driving mechanism discussed before, the sign of the linear driving force depends on the THz electric field's polarity and $F_{\mathrm{dr}}^{\mathrm{IR}}$(ψ = 0°) = $-F_{\mathrm{dr}}^{\mathrm{IR}}$(ψ = 180°). The assumption of a linear driving mechanism is confirmed by the linear scaling of the low‐frequency modes’ amplitude with the THz peak field (Figure 4d). The observation is also consistent with the broad excitation spectrum covering the 0.9 and 1.4 THz frequencies and with complementary THz transmission experiments (see Figure S20, Supporting Information).

Finally, we explain the observed polar pattern by considering the decomposition of the signal amplitude into excitation and detection terms in an IR‐drive‐Raman‐probe type experiment.^[^ ^39^ ^]^ We find the dipole moments of the IR‐active modes to be oriented along the long axis of the flake (ψ = 0°). The driving force is proportional to the projection of the THz electric field along the mode dipole moment, which can be expressed as:

(3) $$\begin{array}{c} F_{\mathrm{dr}}^{\mathrm{IR}}\propto Z^{*}\cdot E^{\mathrm{THz}} \end{array}$$

where $Z^{*}$ is the mode effective charge. Thus, the driving force amplitude follows a dipolar pattern and is maximal for THz parallel to the flake long axis, i.e., ψ = 0°   or 180°. As our detection scheme is only sensitive to Raman‐active modes, the 0.9 and 1.4 THz linearly driven modes must simultaneously be both IR and Raman active.^[^ ^39^ ^]^ We calculate the probing sensitivity for those modes as described previously, using a Raman tensor of the form $R^{\alpha }$. The product of the probing sensitivity and driving force reproduces well the observed azimuthal angle dependence of the signal in Figure 4f. Not only the amplitude (Figure 4e), but also the sign of the theoretically calculated signal (Figure 4f) agrees well with the experimental data, reflecting the π phase jump between neighboring lobes of the polar pattern.

The dominance of linear excitation via linear THz field coupling to the mode's dipole moment (mode effective charge) for modes that are both IR‐ and Raman‐active can be explained by the expected lower efficiency of the nonlinear driving pathway. Crucially, the simultaneous IR and Raman activity of the 0.9 and 1.4 THz modes contradicts the mutual exclusion principle imposed by a globally centrosymmetric crystal structure. We observe the same phase behavior for the low‐frequency cage modes in *n* = 2 and 3 samples (see Section S13, Supporting Information), indicating that globally hidden local or dynamic symmetry breaking is experienced by phonon modes of the inorganic sublattice in the entire RPP family.

## 3 Discussion

Using intense THz fields, we drive coherent lattice dynamics in compounds of the (PEA)_2_(MA)*_n_*_‐1_Pb*_n_* I_3_ *_n_*_+1_ 2D perovskite family. In contrast to BA^+^‐based RPP compounds, which previously showed room temperature coherent lattice responses only for *n* = 1 inorganic layers,^[^ ^10^ ^]^ we observe the excitation of coherent phonons also for the less confined *n  =  *2,3 structures, extending the potential for phonon‐driven material control under application‐relevant conditions. This difference in lattice coherence when employing PEA^+^ and BA^+^ ligands highlights the impact of the organic cation on the global lattice behavior. The highly hydrophobic, bulky PEA^+^ cations improve material stability^[^ ^71^ ^]^ and lead to reduced structural disorder, which is likely the key to the observed extension of phonon coherence times.^[^ ^65^ ^]^ We furthermore identify a strongly anharmonic 2.9 THz mode that is absent in related 3D compounds under the same experimental conditions,^[^ ^7^ ^]^ which we assign to a hybridized organic‐inorganic interface mode and which potentially mediates the organic‐inorganic sublattice coupling and could be used as a handle to target interlayer charge transfer^[^ ^72^ ^]^ in 2D RPPs. Our observations confirm the strong nonlinear response of layered 2D perovskites in the THz range,^[^ ^10^ ^]^ complementing previous reports of exceptionally high nonlinearities in the optical range,^[^ ^44^ ^]^ suggesting application potential for ultrafast THz photonic devices.

We observe multiple anisotropic Raman‐active modes in (PEA)_2_PbI_4_ in the 0.5–3 THz range, and with our systematic polarization‐ and phase‐resolved analysis, we identify their linear and nonlinear excitation conditions, laying the groundwork for selective THz lattice control. Strikingly, the two lowest‐frequency modes at 0.9 and 1.4 THz show simultaneous IR‐ and Raman‐activity. In the closely related (BA)_2_PbI_4_ compound, a THz transmission study found 0.9 and 1.2 THz IR‐active transitions with clear linear in‐plane anisotropy polarized along the same crystal direction, assigned as octahedral rocking modes.^[^ ^73^ ^]^ Given the structural similarity to (PEA)_2_PbI_4_, the two lowest frequency modes observed by us are likely related to analogous inorganic cage dynamics. A local inversion symmetry breaking is a plausible explanation for the intriguing lifting of the mutual exclusion rule. With our broadband single‐cycle THz pulse, covering 0.5–2.5 THz, we expect to efficiently linearly drive all of the IR‐active modes in this range. The simultaneous Raman and IR activity could alternatively be explained by a modification of the optical selection rules by strong spatial electric field gradients on the length scales of atomic bonds, as in the case of surface‐enhanced Raman scattering experiments.^[^ ^74^ ^]^ However, these should not occur under our experimental conditions. The linearly‐ and nonlinearly‐driven modes show similar maximum *S(* ψ) amplitudes in the transient birefringence signal. This seems surprising as signal amplitudes of a resonant effective second‐order nonlinear process are expected to be higher than for a resonant third‐order process.^[^ ^21^, ^75^ ^]^ Nevertheless, this observation is in line with the non‐resonant cases of second harmonic generation (SHG) and third harmonic generation (THG) in (PEA)_2_PbI_4_, where SHG was found to be weaker than THG, which was attributed to the particular form of the anharmonic potential governing the electronic polarizability.^[^ ^21^ ^]^ Similarly, a recent work on the electric‐field‐induced second harmonic generation in (PEA)_2_PbI_4_ reported a dominant role of χ^(3)^,^[^ ^76^ ^]^ indicating that potentially a simple model is insufficient to describe the nonlinear response in this material.

The topic of inversion symmetry breaking in HOIPs has been widely studied using second harmonic generation (SHG) spectroscopy and microscopy.^[^ ^21^, ^77^ ^]^ In PEA^+^‐based RPPs, SHG has been previously observed and attributed to symmetry breaking induced by the ligand molecules.^[^ ^21^ ^]^ However, reports on the presence and absence of SHG in samples of nominally identical composition are often inconsistent. The discrepancies have been assigned to edge states, grain boundaries, and cancellation from random grain orientation.^[^ ^21^ ^]^ These factors should be negligible in our study on highly ordered single crystals. However, we did not observe any SHG in macroscopic experiments using 800 nm pump/400 nm detection on the samples studied here. SHG‐based studies can further be complicated by multiphoton photoluminescence,^[^ ^21^ ^]^ highlighting the advantages of our coherent phonon fingerprint approach to detecting inversion symmetry breaking. While SHG measurements primarily are sensitive to the electronic nonlinear susceptibility, our method directly interrogates these specific ionic motions, which witness an inversion symmetry‐broken lattice potential. Our approach offers an alternative to conventional THz transmission spectroscopy for studying IR‐active modes in non‐centrosymmetric materials.^[^ ^39^ ^]^

The inversion symmetry breaking observed here is unexpected, as most structural studies of *n* = 1 (PEA)_2_PbI_4_ determine a globally centrosymmetric, triclinic $\bar{P1}$ space group.^[^ ^32^, ^47^ ^]^ However, crystallographic studies often provide incomplete or averaged information on the organic cation orientation, potentially leading to conflicting assignments of the same compound to either centrosymmetric or non‐centrosymmetric space groups.^[^ ^21^ ^]^ Two recent works based on combined X‐ray diffraction and DFT analysis proposed a multiconfigurational, polymorphic ground‐state structure in (PEA)_2_PbI_4_, leading to intrinsic inversion symmetry breaking and the presence of the Rashba effect, despite the structure being conventionally assigned as centrosymmetric.^[^ ^32^, ^78^ ^]^ The tilt of the inorganic cages around the c* crystallographic axis and the rotation of the ligand molecules were proposed as degrees of freedom responsible for the emergence of the different phases. Inversion symmetry breaking could emerge at the interfaces between the distinct sub‐phases, each belonging to a centrosymmetric $\;\bar{P1}$ space group,^[^ ^32^ ^]^ or occur in entire crystalline domains due to misalignment of the PEA^+^ cations in adjacent layers.^[^ ^78^ ^]^

Previous studies assigned the *n* = 2 and *n* = 3 compounds from the (PEA)‐based lead iodide family to centrosymmetric $\;\bar{P1}$ ^[^ ^21^, ^79^ ^]^ and noncenstrosymmetric *P*1^[^ ^80^ ^]^ space groups, respectively while second harmonic generation as a marker of inversion symmetry breaking was observed for both of them.^[^ ^21^ ^]^ In our work, the lowest‐energy Pb–I modes serve as dynamic fingerprints of inversion symmetry breaking, independent of the number of the octahedral layers (*n  =  *1–3). This contrasts with mechanisms related to the out‐of‐plane symmetry breaking dependent on an odd/even number of inorganic layers recently proposed for RPPs.^[^ ^26^ ^]^ Thus, we tentatively attribute our observation of hidden symmetry breaking to local rotational distortions of the inorganic cages, present independent of the out‐of‐plane layer stacking and ordering of the layers. In‐plane phonons related to inorganic cage tilting would be natural indicators of such nontrivial, intrinsic symmetry breaking.

In complex structures like layered 2D HOIPs, it is essential to consider symmetries separately at the octahedral cage, inorganic sub‐unit, and on a global level.^[^ ^30^ ^]^ Several recent works proposed the presence of local, as opposed to global inversion symmetry breaking in MHPs.^[^ ^30^, ^31^ ^]^ The presence of local inversion‐symmetry breaking, despite a globally centrosymmetric space group, has been previously linked to structural disorder on the micrometer scale in polycrystalline 3D HOIPs.^[^ ^77^ ^]^ In our highly ordered single crystals, the local symmetry breaking may alternatively also be related to quasi‐1D twin domain boundaries within the RPP layers, arising due to collective alignment of the organic molecules and lead‐halide cage distortions, as recently reported for BA^+^‐based RPPs.^[^ ^81^, ^82^ ^]^ Previously, transient dynamic inversion symmetry breaking mechanisms, related to instantaneous distortions of the lead‐halide local environment,^[^ ^36^, ^83^, ^84^ ^]^ as opposed to static distortions, were proposed in HOIPs. At this point, however, we cannot distinguish between the static or dynamic origin of the observed effect. Given that phonons at frequencies as low as 0.9 THz bear the signatures of the broken inversion symmetry, we expect the symmetry breaking to persist for at least the time of an oscillation cycle, that is > 1 ps. Regardless of its static or dynamic character, the inversion symmetry breaking evidenced in our work is highly consequential for the optoelectronic and spintronic properties of the (PEA)_2_MA_n‐1_Pb_n_I_3n+1_ perovskite family, potentially leading to the emergence of a Rashba effect, chiro‐optical activity, and ferroelectricity.

Exploring the exact microscopic nature and potentially spatially localized character of the hidden inversion symmetry breaking opens an exciting avenue for future research using spatially‐resolved methods such as sum‐frequency microscopy,^[^ ^85^ ^]^ Kerr effect microscopy,^[^ ^86^ ^]^ THz s‐SNOM,^[^ ^87^ ^]^ or piezoresponse force microscopy.^[^ ^88^ ^]^ Composition tuning of the perovskite materials, for example through ligand engineering,^[^ ^89^ ^]^ could be employed to control the inversion symmetry breaking, as even small chemical substitutions in organic molecules may significantly influence the hybrid crystal symmetry.^[^ ^23^ ^]^ Finally, addressing the changes of the vibrational spectrum and phonon mode symmetries under electronic excitation^[^ ^90^, ^91^ ^]^ would constitute a crucial step toward understanding charge carrier‐phonon coupling and polaron formation in MHPs. This can be addressed by future transient birefringence experiments with simultaneous optical injection of charge carriers.

Hidden, local symmetry breaking is potentially intrinsic and highly relevant to a wide range of nominally centrosymmetric materials, in which distinct local subunits may exhibit short‐range inversion symmetry breaking, enabling functionalities forbidden by the global, long‐range symmetry.^[^ ^92^, ^93^ ^]^ Local nano‐ and mesoscale symmetry breaking has been suggested, among others, in inorganic superconducting perovskites,^[^ ^94^ ^]^ cuprates^[^ ^95^ ^]^ or metal oxides like VO_2_.^[^ ^96^ ^]^ Our study demonstrates that analyzing the resonant ionic contributions to the nonlinear susceptibility provides a robust optical tool to investigate symmetry properties of such compounds.

## 4 Conclusion

In conclusion, we demonstrate selective, THz‐driven coherent lattice control in layered PEA^+^‐based RPP compounds with a varying degree of 2D confinement. We identify a vibrational mode specific to the 2D system, which likely mediates the interactions between the inorganic 2D layers and organic spacer molecules. By a systematic polarization study of the THz‐induced transient birefringence, we observe signatures of elusive, globally hidden inversion symmetry breaking, solely experienced by the lowest frequency inorganic cage modes. Coherent control of these vibrational modes could provide a promising handle for influencing RPP optoelectronic and spintronic behavior at ultrafast timescales. Further, spatially‐resolved investigations of the microscopic nature of the hidden symmetry breaking may deliver crucial insights for harnessing emergent properties originating from the material's local or transient non‐centrosymmetric character.

## 5 Experimental Section

### Crystal Synthesis

The single crystal samples were grown by a modified slow‐cooling method described by Bakr and Sargent^[^ ^97^ ^]^ (free‐standing crystals for n = 1,2,3, Figure S1a–c, Supporting Information) or by the Anti‐solvent Vapor‐Assisted Capping Crystallization AVCC method^[^ ^98^ ^]^ (thin crystals on glass substrate for n = 1,2, Figure S1d,e, Supporting Information). In the slow‐cooling method, the solutions were cooled with a 1 °C h^−1^ rate down for *n* = 1 and with a slower rate of 0,12 °C h^−1^ for *n* = 2, 3. The details of the syntheses are described in Ref. [97] and Ref. [98] The regular shape of the crystals allowed us to orient them relative to the field polarization directions. In Figure S1 (Supporting Information), the direction corresponding to the long flake edge, for which $\psi =0^{\circ }$ when the axis is parallel to the THz field polarization direction, is marked.

### THz‐Induced Transient Birefringence Measurements

Single‐cycle THz pump pulses (linearly polarized, peak fields of ≈1 MV cm^−1^, 0.5–3 THz, τ_FWHM_ ≈ 190 fs) used to induce the transient birefringence were generated by optical rectification in LiNbO_3_.^[^ ^99^ ^]^ The LiNbO_3_ crystal was driven by optical/NIR pulses (wavelength 800 nm, pulse duration 35 fs, pulse energy 5 mJ, repetition rate 1 kHz) provided by a Ti:Sapphire amplifier (Coherent Legend Elite Duo) in the tilted pulse front geometry.^[^ ^99^ ^]^ Broadband pulses at 80 MHz repetition rate (wavelength 800 nm, 400 pJ, 20 fs pulse duration, linearly polarized) output from a Ti:Sapphire oscillator (Vitara) were used for probing. The THz pump (focus FWHM  ≈ 300 µm) and probe (focus FWHM  ≈ 50 µm) pulses were collinearly focused on the sample using a parabolic mirror and lens, respectively. A linear translation stage was used to control the temporal delay between the pump and probe pulses. For detection of the fields radiated by the nonlinear polarization, a heterodyne detection scheme was employed, with the residual transmitted probe acting as a local oscillator. The transmitted probe and nonlinear signal pass through a balancing setup consisting of a quarter‐wave plate and half‐wave plate and a Wollaston prism. The Wollaston prism projects the perpendicular vertical and horizontal polarization components of the local oscillator and signal fields onto two separate photodiodes and the differential signal was detected. The polarization of the probe was controlled by using a half‐wave plate and is set to $45^{\circ }$ relative to the fixed vertical THz polarization direction for all of the measurements, apart from the probe polarization scans described in Section S12 (Supporting Information). The room temperature measurements were performed with the sample mounted on a rotating holder inside of nitrogen purging box, and for low‐temperature measurements, the sample was placed inside an optical, liquid nitrogen‐cooled flow cryostat.

### Time‐Domain THz Transmission Spectroscopy

THz pulses generated by optical rectification in LiNbO_3_ (identical to those in the transient birefringence measurements) were focused on the sample, and the transmitted THz field was first collected by a second parabolic mirror, and then subsequently focused into an electro‐optic sampling crystal (100 µm ZnTe), collinearly with an 800 nm probe beam. The same heterodyne detection scheme as described for the TKE experiments was used to record the transient birefringence.

### Raman Spectroscopy

Polarization‐resolved static micro‐Raman measurements were measured using the Horiba T64000 Raman triple grating spectrometer under ambient conditions with a 647 nm excitation laser in backscattering geometry. The incident beam was linearly polarized perpendicular to the 2D perovskite layer planes, and the polarization direction relative to the sample controlled using a half‐waveplate. The scattered light was recorded by a spectrometer after passing through a linear polarization analyser, allowing to separately measure the co‐ and cross‐polarized contributions.

## Conflict of Interest

The authors declare no conflict of interest.

## Supporting information



Supporting Information

## Data Availability

The data that support the findings of this study are available from the corresponding author upon reasonable request.
