# Supplementary material for: THz‐Driven Coherent Phonon Fingerprints of Hidden Symmetry Breaking in 2D Layered Hybrid Perovskites
Source: Adv Mater. 2025 Oct 3;38(4):e02204. doi: 10.1002/adma.202502204 (PMC12810635; doi:10.1002/adma.202502204)
Supplement: Supplementary file 1 — Supporting Information [file ADMA-38-e02204-s001.pdf]

ADVANCED MATERIALS

Supporting Information

for *Adv. Mater.*, DOI 10.1002/adma.202502204

THz-Driven Coherent Phonon Fingerprints of Hidden Symmetry Breaking in 2D Layered Hybrid Perovskites

Joanna M. Urban, Michael S. Spencer, Maximilian Frenzel, Gaëlle Trippé-Allard, Marie Cherasse, Charlotte Berrezueta-Palacios, Prakriti P. Joshi, Alexander P. Fellows, Olga Minakova, Eduardo B. Barros, Luca Perfetti, Stephanie Reich, Martin Wolf, Emmanuelle Deleporte and Sebastian F. Maehrlein**

Supporting Information

Supporting Information for

THz-Driven Coherent Phonon Fingerprints of Hidden Symmetry Breaking in 2D Layered Hybrid Perovskites

Joanna M. Urban, Michael S. Spencer, Maximilian Frenzel, Gaëlle Trippé- Allard, Marie Cherasse‡, Charlotte Berrezueta-Palacios, Prakriti P. Joshi, Alexander P. Fellows, Olga Minakova, Eduardo B. Barros, Luca Perfetti, Stephanie Reich, Martin Wolf, Emmanuelle Deleporte, Sebastian F. Maehrlein**

Section S1. <u>Sample growth and morphology</u>

The samples were grown as free-standing crystals by the slow-cooling method[1] as well as, for n=1,2, by the Anti-solvent Vapor-Assisted Capping Crystallization (AVCC) method[2] on BK7 glass substrates. The AVCC crystals were measured as-grown on the substrate (**Figure S1 d-e**)) and the slow-cooling crystals either as-grown (**Figure S1 a-c**) or after thinning down by scotch tape cleaving. The regular shape of the crystals allowed us to orient them relative to the field polarization directions. In Figure S1 d, e, the direction corresponding to the long axis of the AVCC crystals, defining the azimuthal angle $\psi = 0^{\circ}$ when parallel to the THz field polarization direction, is marked. For the slow-cooling crystals, the short/long edge assignment was more ambiguous but it was possible to distinguish between directions parallel and diagonal to the crystal edges.

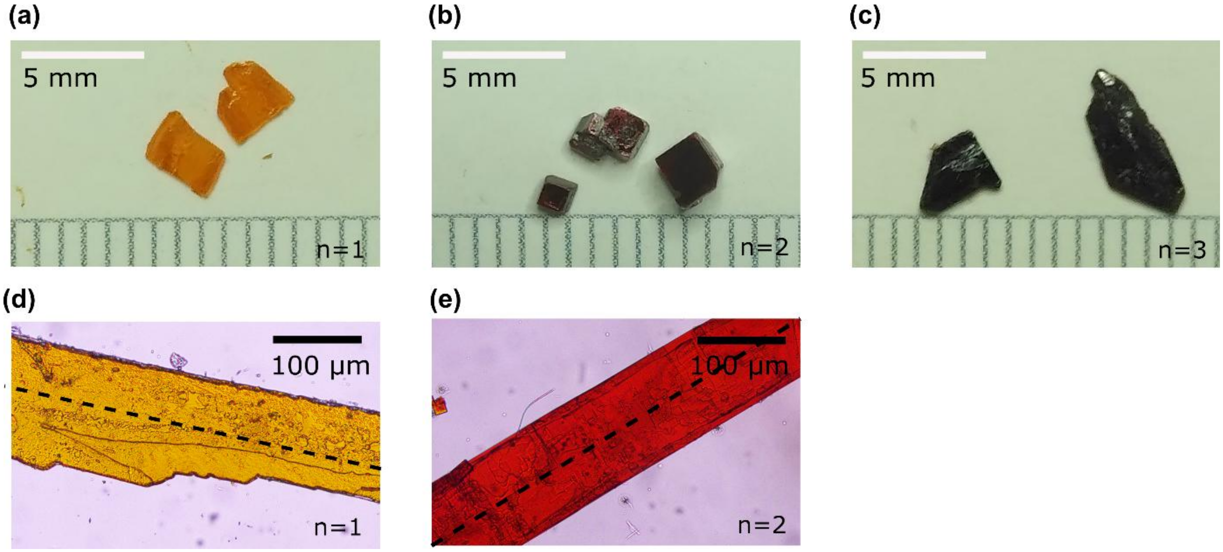

Figure S1. Optical images of **a)** slow-cooling *n*=1, **b)** slow-cooling *n*=2, **c)** slow-cooling *n*=3, **d)** AVCC *n*=1, **e)** AVCC *n*=2 crystals. The dashed line marks the flake long axis.

Section S2. Influence of sample thickness on transient birefringence signal

To exclude possible misinterpretation of the oscillatory transient birefringence signals due to propagation effects,[3,4] measurements for the n=1 compound were performed on multiple samples with different thicknesses. **Figure S2** shows a comparison of the room temperature signals measured on a very thin (d < 1 μm) AVCC crystal on BK7 substrate and on an as-grown free-standing slow-cooling crystal of d ~230 μm thickness. Features related to propagation artifacts should be strongly thickness dependent, so the observation of very similar peaks in the spectra of the two different samples in **Figure S2b** (0.8-0.95 THz, 1.15-1.35 THz and 1.55-1.65 THz) confirms their assignment as coherent phonon signatures. Especially the ~0.9 THz mode clearly dominates the signals for longer cutoff times (**Figure S2c**) for both samples. A smearing of the instantaneous response features in time domain is visible for the thicker sample in the form of a slight exponential tail.[3] Slight differences between the spectra of the two samples observed at shorter cut-off times (**Figure S2b**) may be related to interferences of the lattice response with residual contributions from the temporally smeared out instantaneous nonresonant response in the case of the thicker sample.

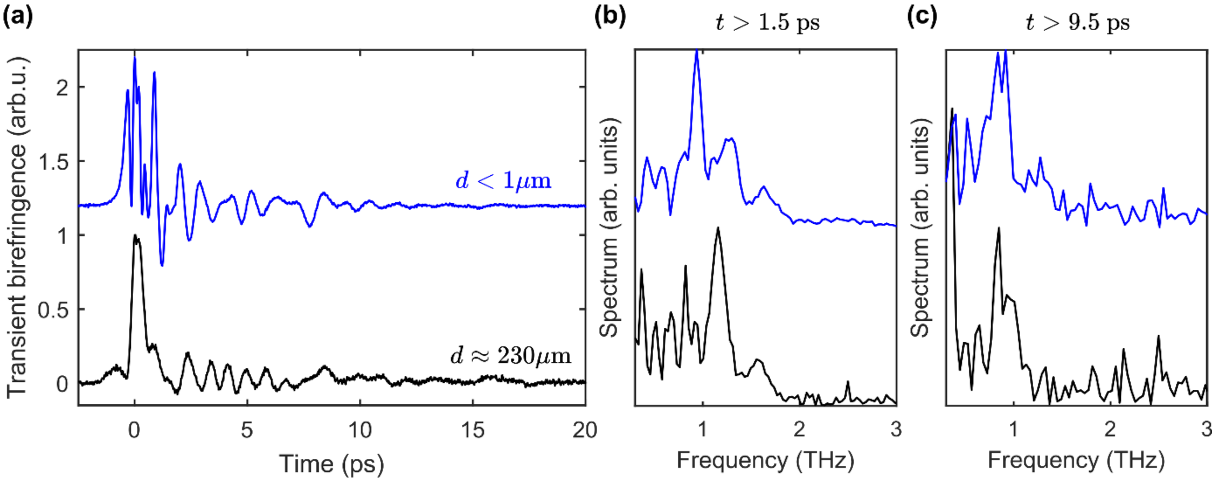

Figure S2. **a)** Transient birefringence signal recorded for a thin AVCC (blue) and thick slow-cooling (black) crystals at = 0° . **b)** Corresponding FTs of the oscillatory signal for cut-off time 1.5 ps and **c)** 9.5 ps.

Section S3. Sample thickness: pump-probe walk-off effects

We note differences between the relative intensities of the different phonon signatures in the signals measured on different samples, with the higher frequency modes typically becoming less prominent in thicker slow-cooling samples compared to thinner AVCC crystals. **Figure S3a** compares the FTs of transient birefringence signals measured on four different samples of n=1, thin AVCC (typically d < 5 μm), a thick AVCC sample and a slow-cooling sample (typically d > 50 μm). The walk-off effects due to different propagation velocity of the pump and probe pulse[5] will lead to a relative decrease of the higher-frequency components of the driving force at finite depths in the sample, as demonstrated by simple modelling in **Figure S3 b, c**. For simplicity, we consider only the real part of the refractive index, neglecting absorption

and anisotropy, and assume $n_{\mathrm{THz}} = 2.9$ (calculated from approximate $\tilde{\varepsilon}_{\infty}$ based on data from ref.[6]) and $n_{800} = 2$ (extrapolating after[7]). The incident field is extracted from an electro-optic sampling measurement after detector response function correction.[8] The driving force spectrum for the photonic driving mechanism is proportional to the Fourier transform of the squared THz field:

$$F_{\mathrm{dr}}(\omega) \propto \mathrm{FT}\left(E_{\mathrm{THz}}^{2}(t)\right) \qquad (S1)$$

The THz-induced Kerr-type transient birefringence signal $S(t)$ related to instantaneous electronic polarizability can be written as being proportional to:[9]

$$S(t) \propto \int_{0}^{d} dz\, \Delta n\left(z, t + \frac{z}{v_{800}}\right) = \int_{0}^{d} dz\, \Delta n(0, t + \beta z) \propto \int_{0}^{d} dz\, F_{\mathrm{dr}}(0, t + \beta z) \qquad (S2)$$

where $\beta = v_{800}^{-1} - v_{\mathrm{THz}}^{-1} = (n_{800} - n_{\mathrm{THz}})c_0$ is the inverse velocity mismatch between the probe and the pump and $\Delta n(z,t) = n_2 c_0 \varepsilon_0 E_{\mathrm{THz}}^{2}(z,t) \propto F_{\mathrm{dr}}(z,t)$ is the THz-induced refractive index anisotropy determined by the square of the THz electric field and the nonlinear refractive index n_2, assuming the THz field after propagating a distance z inside the sample can be written as $E_{\mathrm{THz}}(z,t) = E_{\mathrm{THz}}(z = 0, t - z v_{\mathrm{THz}}^{-1})$ [9] and neglecting THz absorption. In frequency domain:

$$S(\omega) \propto \mathrm{FT}\left(\frac{1}{\beta} F_{\mathrm{dr}}(0,t) * \mathrm{rect}\left(\frac{t}{\beta d} - \frac{1}{2}\right)\right)$$
$$\propto \mathrm{FT}\left(E_{\mathrm{THz}}^{2}(0,t)\right) \cdot d \cdot \exp\left(-\frac{i\omega\beta d}{2}\right) \mathrm{sinc}\left(\frac{\omega\beta d}{2}\right) = F_{\mathrm{eff,dr}}(\omega, d) \qquad (S3)$$

where the last expression describes a modified effective driving force spectrum $F_{\mathrm{eff,dr}}(\omega, d)$ resulting from integration over the sample thickness. Figure S3b shows the effective driving force spectrum as a function of the sample thickness compared to the driving force calculated for the field at $z = 0$. In frequency domain (Figure S3b), the driving force spectral amplitude bandwidth decreases significantly with increasing sample thickness. In time domain (Figure S3c), the velocity mismatch leads to a smearing out of the electronic response signal and appearance of box-like signals for larger thicknesses.[9] Including anisotropy, the dispersion of the THz refractive index and absorption which are both significant in $(PEA)_2PbI_4$ in the 0-5 THz range (see Section S4) to fully describe our system would result in a significantly more complex response, but maintaining a similar driving force bandwidth reduction for thicker samples.

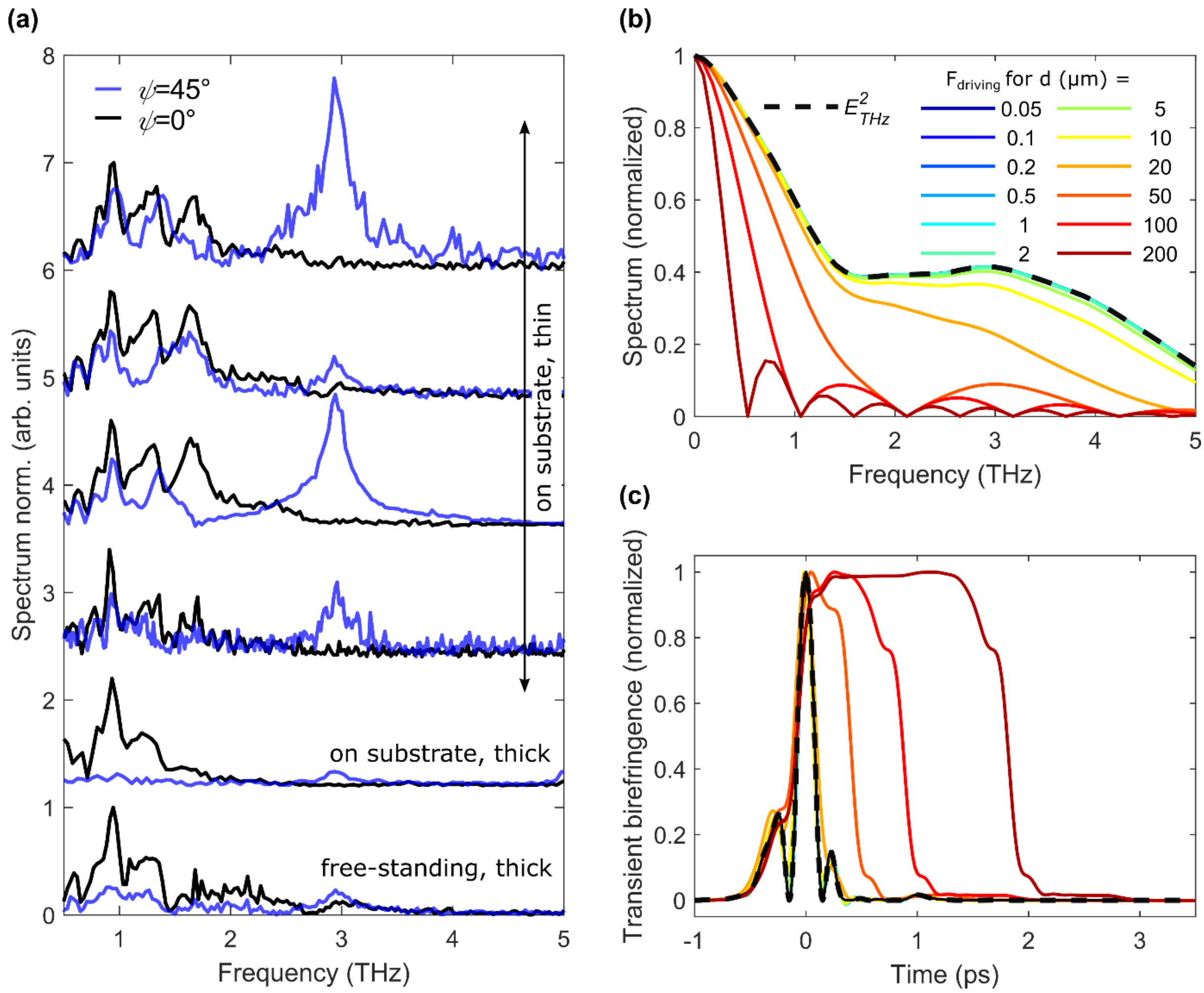

Figure S3 a) Fourier transforms of transient birefringence spectra for sample azimuthal orientations $\psi = 0^{\circ}$ and $\psi = 45^{\circ}$ for thin and thick AVCC crystals and thick slow-cooling free-standing crystals. Each pair of spectra for the two angles for each sample were normalized by the intensity of the 0.9 THz peak for $\psi = 0^{\circ}$. **b)** Driving force spectrum calculated based on the incident field (black, dashed) and considering walk-off effects for a range of thicknesses *d*. **c)** Corresponding time domain TKE instantaneous response signals.

Section S4. Absorption and dispersion effects

Figure S4a shows the complex refractive index terms for $(PEA)_2PbI_4$ calculated from the dielectric constant given in Ref.[6]. The frequency-dependent penetration depth of the THz into the sample can be estimated based on the absorption coefficient as (**Figure S4b**):

$$l = \alpha^{-1} = \frac{\lambda_0}{4\pi k} \qquad (S4)$$

Figure S4c shows the modification of the spectrum of the THz field and corresponding driving force upon transmission into the sample taking into account the transmission coefficient at the air-sample interface:

$$t = \frac{2}{(\tilde{n}_{\mathrm{THz}} + 1)} \qquad (S5)$$

Figure S4d shows the spectra of the THz electric field and the driving force after propagation for different finite depths into the sample. Higher frequencies are more strongly absorbed, leading to a reduction of the field bandwidth for larger thicknesses. Together with propagation effects, absorption could explain the higher contribution from higher frequency modes in thinner samples.

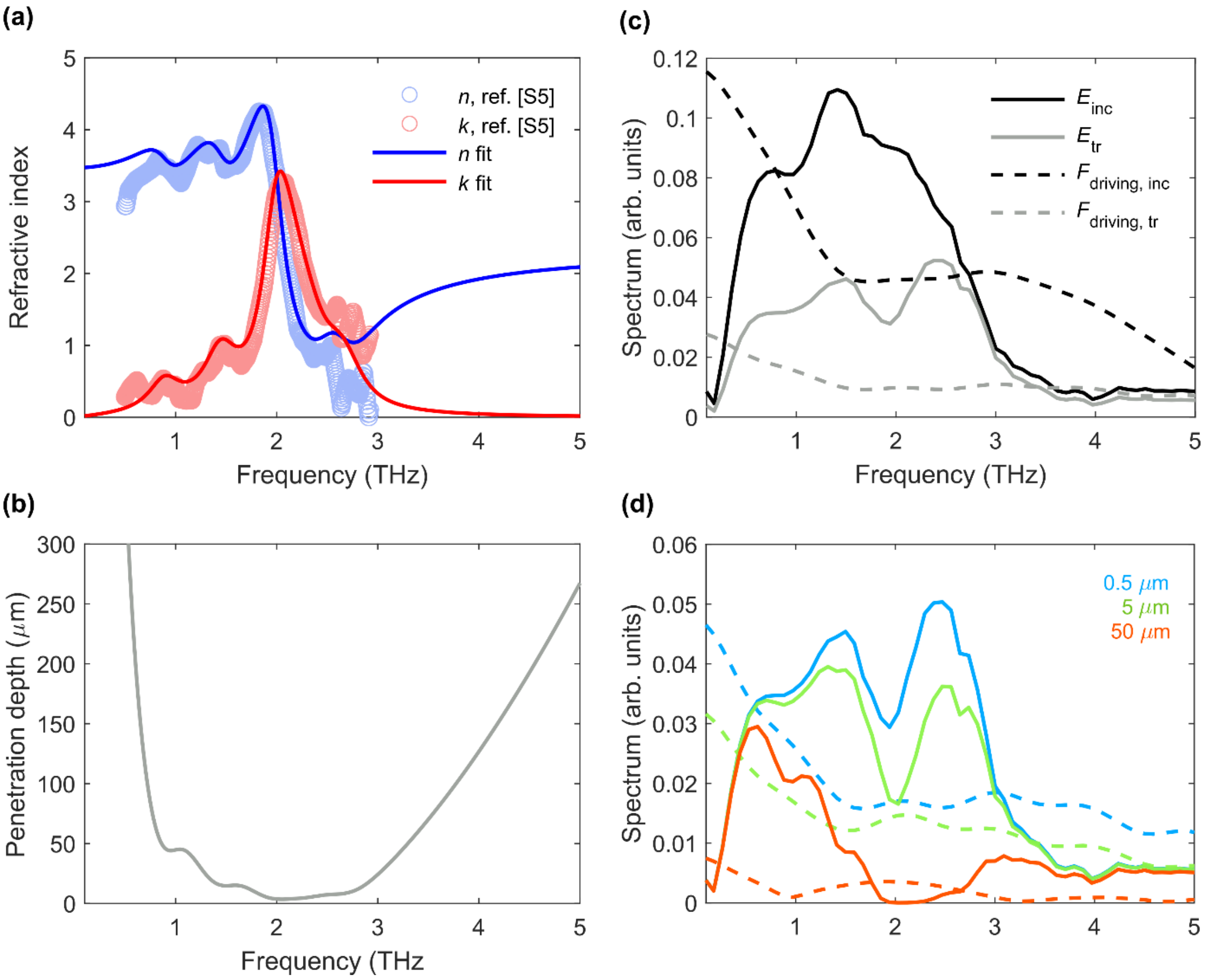

Figure S4 a) Complex refractive index $\tilde{n} = n + ik$ calculated based on the dielectric function from ref. [6] and its fits using three Lorentzian oscillators. **b)** Penetration depth in the THz range calculated based on k. **c)** The incident THz electric field spectrum E_{inc}, the field transmitted through the air-sample interface E_{tr} and the respective nonlinear driving force spectra. **d)** Electric field spectra and driving force spectra at a depth z =0.5, 5 and 50 μm in the sample.

Section S5. Estimation of nonlinear refractive index

The nonlinear refractive index at ~1 THz is estimated for the n=1 sample by converting the measured transient birefringence signal into a rotation angle (**Figure S5**) and extracting the instantaneous peak rotation $\Delta\phi_{max}$ = 31.1 mrad.

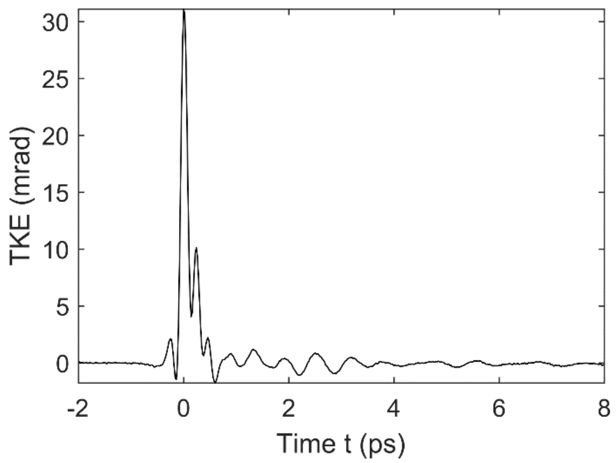

Figure S5 Transient birefringence signal converted into rotation angle for n=1 AVCC sample at $\psi = 0^{\circ}$.

Assuming $n_{1\text{THz}} = 3.51$ and $k_{1\text{THz}} = 0.53$ (see Figure S4a), a thickness of the sample $d = 5\mu\text{m}$, an absorption coefficient $\alpha_{1\text{THz}} = 4\pi k_{1\text{THz}}\lambda_{1\text{THz}}^{-1} = 2235.5$ cm^{-1}, an incident THz peak electric field $E_{peak} = 1.1$ MV/cm and an air-sample transmission coefficient given by Eq. (S5) $t_{1\text{THz}}$, we can write a ballpark estimate for n_2:[3,9]

$$n_2(1\text{ THz}) = \frac{\Delta\phi_{max} \cdot \lambda_{800}}{2\pi c_0 \varepsilon_0 \int_0^d dz (E_{1\text{THz}}(z))^2} =$$

$$= \frac{\Delta\phi_{max} \cdot \lambda_{800}}{2\pi c_0 \varepsilon_0 \int_0^d dz\, (t_{1\text{THz}} \cdot E_{peak} \cdot \exp(-0.5 \cdot \alpha_{1\text{THz}} \cdot z))^2} \quad \text{(S6)}$$

which gives a value of $n_2(1\text{THz}) \approx 1.4 \cdot 10^{-12}$ cm/W. The main source of uncertainty in the estimate of n_2 is the unknown exact sample thickness. The typical thickness of AVCC crystals is in the 0.5-10 μm range. Assuming 0.5 μm and 10 μm thicknesses for the calculations yields upper and lower limits for the estimated values of $n_2(1\text{THz}) \approx 1.3 \cdot 10^{-11}$ and $n_2(1\text{THz}) \approx 7.2 \cdot 10^{-13}$, respectively.

Section S6. Probe fluence dependence

We measured the transient birefringence signals as a function of varying probe fluence for the *n*=1 sample at two different orientations (long flake axis at $\psi = 0^{\circ}$ and $\psi = 45^{\circ}$ to THz polarization) to confirm that there are no additional effects caused by the presence of charge carriers generated by two-photon absorption and that the signal scales according to our predictions. The normalized signals (**Figure S5a** and **b**) and the corresponding FTs (**Figure S5c** and **d**) do not show any changes with the probe fluence. In the four wave mixing picture, the nonlinear signal field $E_{\text{signal}}^{(3)}$ is proportional to the probe electric field at the depth z $E_{\text{probe}}(z)$ in the sample where the signal is generated. Assuming that most of the signal is generated near the surface of incidence, we can assume $E_{\text{probe}}(z) \approx E_{\text{probe,incident}} \sim \sqrt{I_{\text{probe}}}$ where I_{probe} is the measured power of the incident probe. The signal measured in our detection

scheme assuming balanced conditions is proportional to $(E^{(3)}_{\mathrm{signal,x}} - E^{(3)}_{\mathrm{signal,y}}) \cdot E_{\mathrm{probe,tr}}$, where $E_{\mathrm{probe,tr}}$ is the transmitted probe field after the sample used for heterodyning. The transmitted field can experience losses due to scattering and two-photon absorption, therefore is not simply proportional to $E_{\mathrm{probe,incident}}$.We measure the photodiode voltage V_{PD} on a single channel under balanced conditions for every I_{probe}, assuming that $E_{\mathrm{probe,tr}} \sim V_{\mathrm{PD}}$. We finally plot the transient birefringence signal magnitude as a function of $\sqrt{I_{\mathrm{probe}} \cdot V_{\mathrm{PD}}}$, and obtain almost perfect linear scaling as expected (**Figure S5e** and **f**).

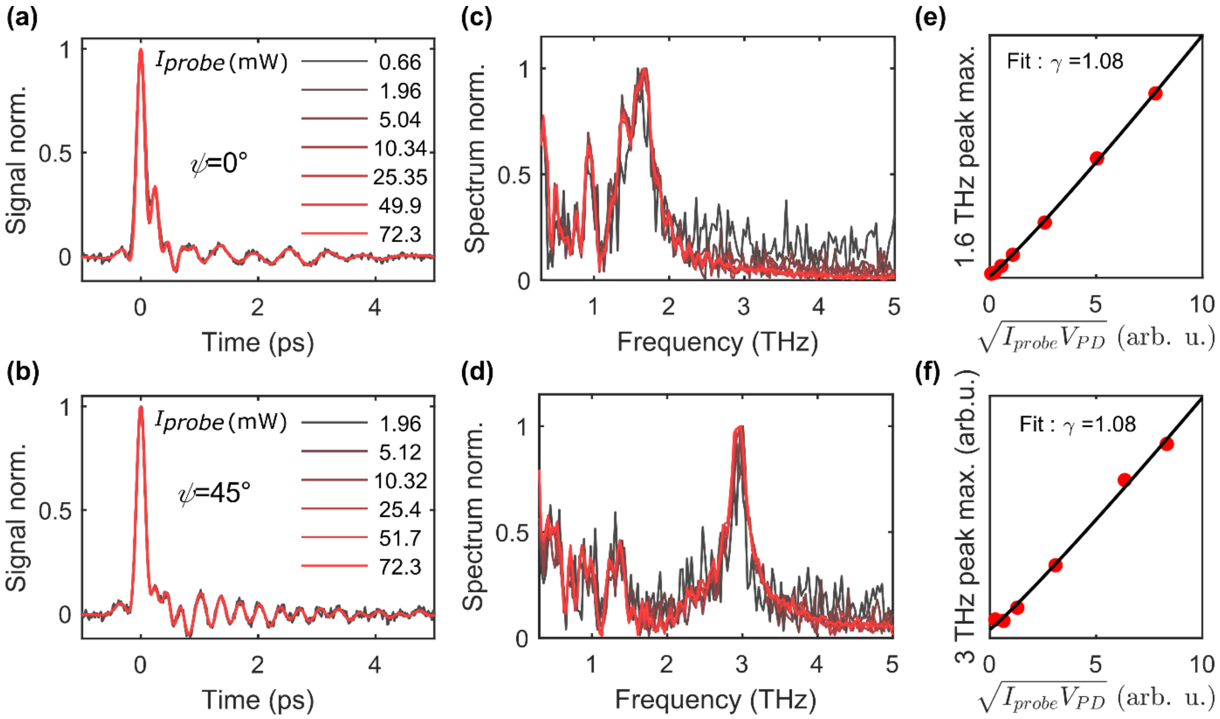

Figure S6. Normalized transient birefringence signals as a function of incident probe power for **a)** $\psi = 0^{\circ}$ and **b)** $\psi = 45^{\circ}$ and corresponding FTs **c)** and **d)**. Amplitude of the **e)** 1.6 THz peak and **f)** 3 THz peak as a function of $\sqrt{I_{\mathrm{probe}} \cdot V_{\mathrm{PD}}}$ and the fitted power laws $A = (\sqrt{I_{\mathrm{probe}} \cdot V_{\mathrm{PD}}})^{\gamma}$.

<u>**Section S7.** Reproducibility of azimuthal angle scans</u>

We repeated the transient birefringence measurement as a function of the crystal azimuthal angle on several flakes to confirm the observed polar patterns. **Figure S7** shows a comparison of the angle-dependent instantaneous response amplitude and the Raman-active mode signal amplitudes for two different flakes in **a** and **b**. In both data sets, we observe deviations of the experimental signal from a four-fold symmetry for the instantaneous response as well as from the theoretically predicted coherent phonon signal amplitude. The differing deviations for the two different flakes suggest that these are experimental artifacts arising due to sample inhomogeneities and/or imperfect sample orientation in the plane normal to the beam.

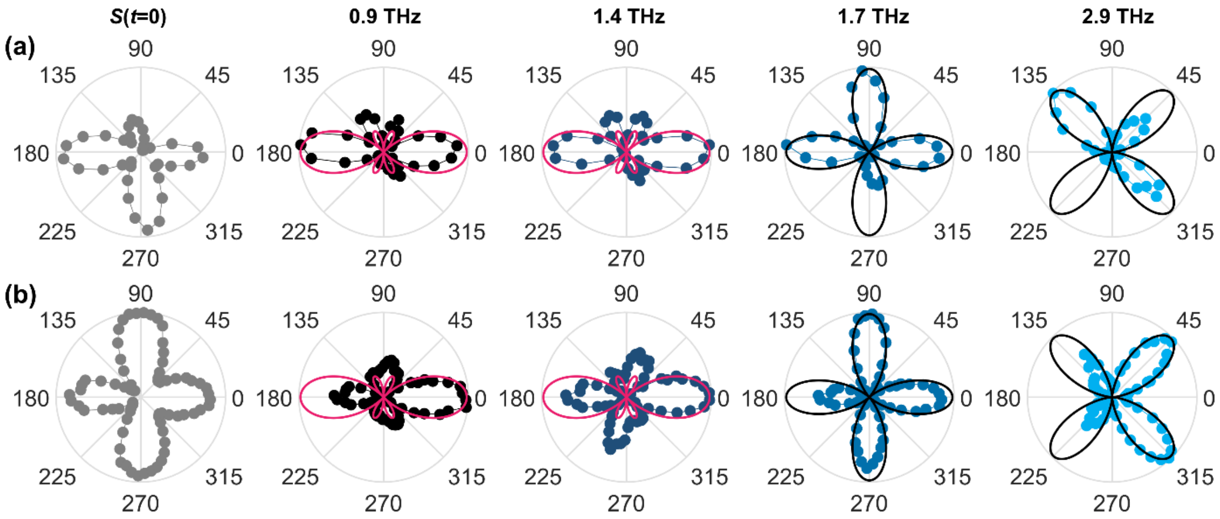

Figure S7. Instantaneous response amplitude $S(t = 0)$, and mode amplitudes extracted from the Fourier transforms of the transient birefringence traces as a function of the sample azimuthal angle for two different *n*=1 flakes in **a)** and **b)**. In both cases, $\psi = 0^{\circ}$ corresponds to long crystal axis parallel to the THz polarization. The magenta and black lines show the theoretically calculated coherent phonon signal amplitude assuming IR driving and Raman probing, or Raman driving and Raman probing, respectively.

<u>Section S8.</u> <u>Balancing artifacts in azimuthal angle scans</u>

Due to our experimental configuration, full azimuthal angle scans could not be performed while adjusting the balancing optics (quarter- and half-wave plate) in the detection setup for every angle ψ. Balancing was therefore done for $\psi = 0^{\circ}$ and the positions of the balancing optics were left unchanged during the scan. Intrinsic sample birefringence in the 800 nm range may lead to slight changes in balancing conditions with azimuthal angle, which in turn could influence the signal amplitude. We estimate the magnitude of this effect comparing a scan where the configuration of the balancing optics was left unchanged from balancing at $\psi = 0^{\circ}$ and a measurement where for each ψ, the balancing was adjusted manually. **Figure S8a** shows the non-normalized signals acquired in both configurations. **Figure S8b** compares the signal Fourier transforms for signals divided by the sum of the voltage on the two diode channels, which was acquired for every angle ψ for each channel, and normalized so that the instantaneous signal amplitude is equal for the balanced and the unbalanced scan at $\psi = 0^{\circ}$. Figure S8 (c) compares the azimuthal angle dependence of the instantaneous peak amplitude (normalized to be equal for both scans at $\psi = 0^{\circ}$) and the FT spectral amplitude at the peaks at around 0.9 THz, 1.7 THz and 2.9 THz. The differences are negligible and do not influence our mode symmetry analysis and conclusions.

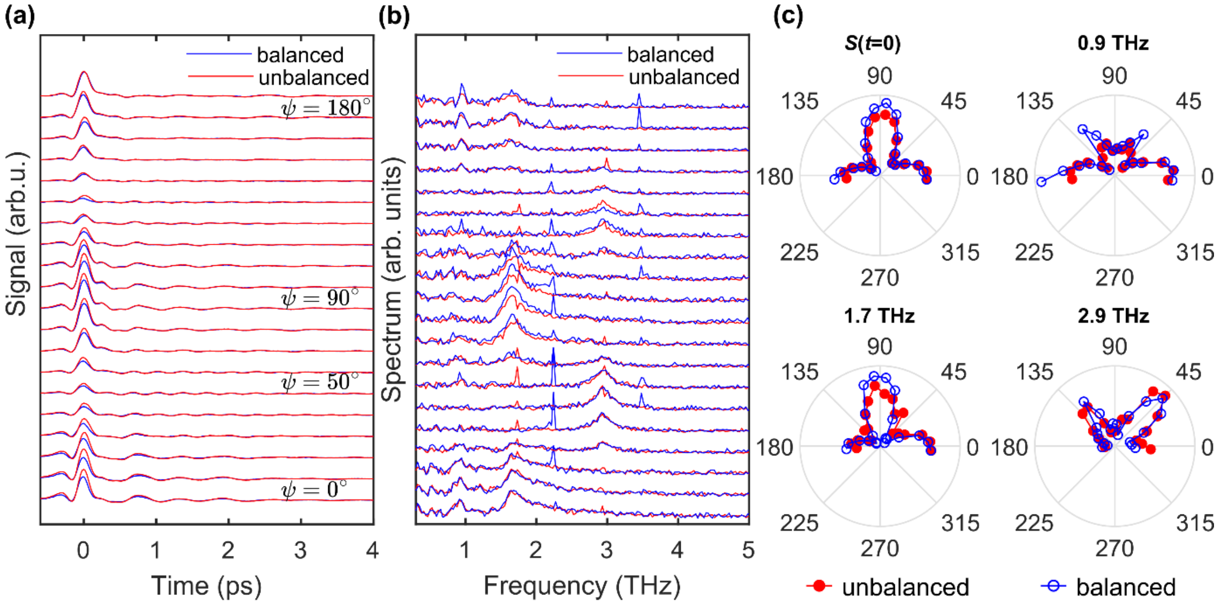

Figure S8. **a)** Transient birefringence signals for different sample azimuthal angles at 10° intervals measured with manual rebalancing at every angle (blue) and without (red). **b)** Corresponding FTs of the oscillatory part of the traces, normalized to match the instantaneous response magnitude at $\psi = 0^\circ$ between the traces**. c)** Magnitude of the instantaneous response peak and the FT spectral amplitude at the 0.9 THz, 1.7 THz and 2.9 THz dominant modes as a function of azimuthal angle extracted from the measurements with and without rebalancing.

Section S9. Phonon lifetime estimation

We estimate the phonon lifetimes based on the linewidths extracted from frequency-domain fits of Lorentzian functions to the respective peaks in the squared Fourier transform amplitude spectrum. As a cross-check we compared time- and frequency-domain fits. **Figure S9a** shows the fitting of the oscillatory signal in time domain with an exponentially damped sine function:

$$S(t) = A \cdot \exp(-\zeta_A t) \cdot \sin(2\pi f_0 t + \varphi) + B \tag{S7}$$

for an exemplary trace acquired on the n=1 sample at 150 K for the azimuthal angle $\psi = 45^\circ$, after substracting an exponential tail related to the instantaneous response and propagation effects. We assume here that the amplitude decay time $\tau_A = \zeta^{-1}$ corresponds to twice the phonon lifetime τ (assuming the absence of elastic scattering leading to pure dephasing), $\tau_A = 2\tau$. From the time domain fit, we extract the frequency and lifetime of the single mode as $f_0 = 3.062 \pm 0.004$ THz and $\tau = 1.9 \pm 0.2$ ps. Figure S9 (b) shows fitting of the squared Fourier transform spectral amplitude of the same trace with a single Lorentzian function:

$$S(f) = C \cdot \frac{\frac{\Gamma}{2}}{(f - f_0)^2 + (\Gamma/2)^2} + D, \tag{S8}$$

where Γ corresponds to the peaks' full width at half maximum. Phonon lifetime is given by $\tau = 1/(2\pi\Gamma)$. From this fit, we obtain the mode frequency $f_0 = 3.066 \pm 0.003$ THz and a phonon lifetime of $\tau = 2.0 \pm 0.2$ ps. The results between time and frequency domain fitting agree very

well, as expected when the signal amplitude fully decays within the measurement window in time domain. We therefore use only the frequency domain fitting for the remaining analysis.

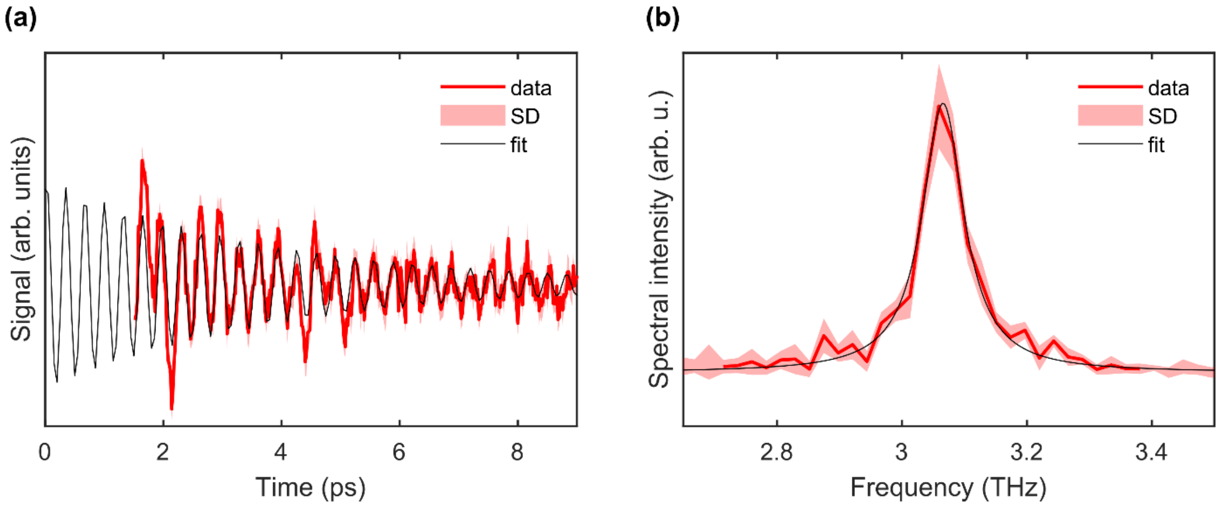

Figure S9. **a)** Oscillatory part of transient birefringence signal for *n*=1 sample at 150 K for the azimuthal angle $\psi = 45^{\circ}$ (red) and damped sine function fit (black). The red shaded area corresponds to the standard deviation of the experimental data. **b)** Corresponding intensity spectrum (squared FT spectral amplitude) and Lorentzian fit in the range of interest.

Figure S10 shows exemplary frequency-domain fits of the low-frequency modes for *n*=1 at $\psi = 0^{\circ}$ at (a) 80 K and (b) 150 K. We use a sum of three Lorentzian functions to fit the spetra in the 0.5-2 THz range. At 80 K, the lowest frequency peak shows a slight splitting, which we do not take into account for the purpose of our analysis.

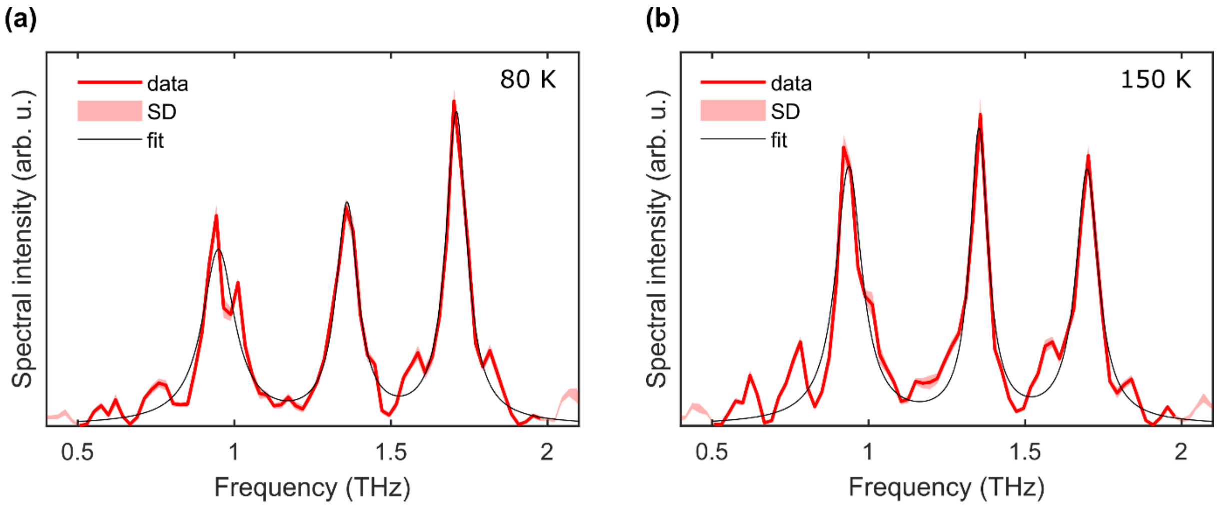

Figure S10. Intensity spectra for the *n*=1 sample at $\psi = 0^{\circ}$ and fits of the three low-frequency phonon modes at **a)** 80 K and **b)** 150 K.

Figure S11 (b) illustrates the relative differences in mode lifetimes by comparing FT amplitude spectra of two room temperature transient birefringence traces for the *n*=1 sample at $\psi = 0^{\circ}$ and $\psi = 45^{\circ}$ (panel **a**), taken after cutting off the data at different pump-probe delay times. For

$t > 0.5$ ps, the low-frequency mode lineshape for $\psi = 0^{\circ}$ is distorted and resembles a Fano profile, due to the interference of the resonance and nonresonant terms in the nonlinear signal[10] (originating from the ionic and instantaneous electronic response, respectively). For $t > 1.5$ ps, the interference effects disappear and both low-frequency modes and the 2.9 THz mode are still detectable in the signal, while at longer times $t > 5$ ps only the low-frequency cage modes contribute to the oscillatory response.

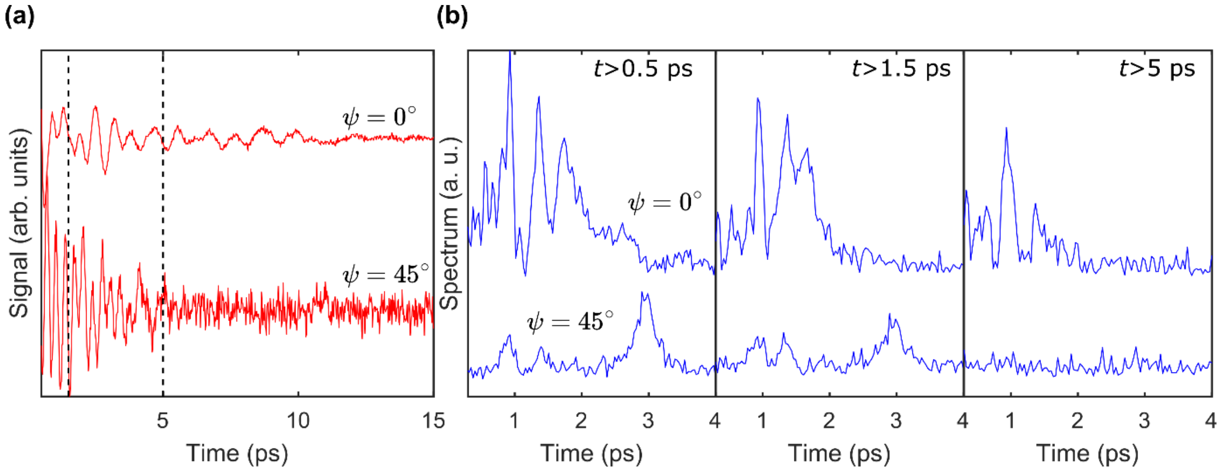

Figure S11. **a)** Transient birefringence signal of the *n*=1 sample at different azimuthal angles. Dashed lines mark the cutoff times. **b)** Corresponding FT spectral amplitude of the traces cut off at different times for the two azimuthal angles.

Section S10. Temperature dependence of phonon lifetime and frequency

Phonon lifetimes and frequencies are obtained based on fitting a Lorentzian function to the norm-squared of the FT amplitude in frequency domain. We assume that the observed decay of the coherent phonon population is mainly governed by inelastic defect-phonon and phonon-phonon scattering, and not by pure dephasing mechanisms, such as electron-phonon (elastic) scattering.[11] In this case, the inverse of the FWHM of the peak for a particular mode gives a good approximation of its lifetime.[11–13] We compare the observed shift and broadening of the phonon modes as a function of temperature T with predictions of a model which considers only the simplest case of a symmetric 3-phonon anharmonic decay.[14,15] The shift of a phonon mode with temperature in this approximation is the sum of the term related to lattice expansion $\Delta f_0(T)$ as well as the one due to the phonon-phonon scattering $\Delta f_{\mathrm{ph},3}(T)$:[14]

$$\Delta f(T) = \Delta f_0(T) + \Delta f_{\mathrm{ph},3}(T), \tag{S9}$$

$$\Delta f_{\mathrm{ph},3}(T) = A\left(1 + \frac{2}{e^{\frac{hf_0}{2k_B T}} - 1}\right). \tag{S10}$$

A is the anharmonic constant, k_B the Boltzmann constant, h the Planck constant and f_0 the phonon frequency in the harmonic approximation at 0 K. We neglect the influence of lattice expansion on the mode frequencies as we do not have detailed information on the evolution of the lattice constants, and also given that the modes have largely in-plane character and the in-

plane lattice expansion as a function of temperature in 2D layered perovskites is not significant and strikingly weaker than in the out-of-plane direction.[16] We therefore fit the observed shift using only the second term in expression (1) and obtain the following parameters (**Table S1**):

Mode	0.9 THz	1.4 THz	1.7 THz	3 THz
A [THz]	-0.00010	-0.00014	-0.00029	-0.0036
f_0[THz]	0.94	1.37	1.72	3.15

Table S1. Parameters obtained from the phonon frequency shift fitting.

The same 3-phonon scattering model predicts the evolution of the phonon linewidth (and corresponding lifetime) as a function of temperature:

$$\Gamma_3(T) = \Gamma_0 + B\left(1 + \frac{2}{e^{\frac{hf}{2k_\mathrm{B}T}} - 1}\right) \qquad \text{(S11)}$$

For the $\Gamma(T)$ fitting, we can assume the frequency $f = f_0$ as a constant parameter equal to the un-renormalized frequency f_0 at 0 K, as the change of f with temperature is relatively small. Γ_0 is the temperature-independent broadening due to impurity scattering,[17] and the anharmonic constant B corresponds to the intrinsic linewidth at $T = 0$. We performed this analysis only for the mode at around 3 THz, as the other modes do not present a clear enough dependence. The fitting parameters obtained are given in **Table S2**:

Mode	3 THz
B [THz]	0.0023
f_0[THz]	3.16
Γ_0[THz]	0.017

Table S2. The parameters obtained from the phonon linewidth fitting.

While in reality, contributions from higher order and asymmetric scattering processes,[18,19] as well as corrections due to lattice expansion may be non-negligible, the simple model qualitatively captures the observed mode behavior. We do not assign the phonon branches into which the original optical modes scatter, which could in principle either be both acoustic (for example the symmetric Klemens mechanism[15]) and optical, as we lack information on the full phonon dispersion of the complex RPP material.

Section S11. Raman tensor assignment and probe polarization dependence

We base our Raman tensor element assignment for the 1.4, 1.7 and 2.9 THz modes on fitting the polarized spontaneous Raman scattering data (see **Section S14**). We only consider the in-plane components of the incident and scattered electric fields, which propagate along *z* and perpendicular to the sample *xy* plane, and therefore assume a 2 x 2 form of the Raman tensor within the *xy* plane. Using the same tensors to describe the probing process and their real parts to describe the driving process allows us to well reproduce the transient birefringence

experimental data. **Table S3** summarizes the Raman tensor assignment and the driving mechanisms of the different modes. Note that as given in Table S3 describe only the general form of the tensors, and to reproduce the quantitative results a scaling factor multiplying the tensor should be used to account for different intensities of the modes. For the 0.9 THz mode we assume the same form of the Raman tensor as for the 1.7 THz mode, as its low intensity in spontaneous Raman scattering hinders precise fitting of the tensor elements.
To confirm the assignment of the mode symmetries, we measured the transient birefringence signal for the *n*=1 sample as function of the probe polarization direction (changed by rotating a half-wave plate in the incoming probe beam) for three different, fixed flake orientations, as shown in **Figure S12**. To calculate the signal, we assigned Raman symmetries and driving mechanisms as summarized in Table S3. Comparing the experimental signal of the four dominant vibrational modes (taking into account both amplitude and sign) with theoretical calculations we obtain very good agreement, as can be seen in **Figure S12 c, f, i.** The very weak signal of the 0.9 THz mode, observed for the flake oriented perpendicular to the THz polarization (Figure S12f), may be present due to imperfect polarization and flake orientation, or represent a weak contribution from the Raman-type driving, not taken into account in our calculation.

Mode	0.9 THz	1.4 THz	1.7 THz	2.9 THz
Driving	IR	IR	Raman	Raman
Raman tensor	$\mathbf{R} = a\begin{bmatrix} 1 & \frac{4}{3}e^{i\pi/2} \\ \frac{4}{3}e^{i\pi/2} & -1 \end{bmatrix}$	$\mathbf{R} = a\begin{bmatrix} 1 & e^{i\pi/2} \\ e^{i\pi/2} & -1 \end{bmatrix}$	$\mathbf{R} = a\begin{bmatrix} 1 & \frac{4}{3}e^{i\pi/2} \\ \frac{4}{3}e^{i\pi/2} & -1 \end{bmatrix}$	$\mathbf{R} = b\begin{bmatrix} e^{i\pi/2} & -\frac{8}{9} \\ -\frac{8}{9} & -e^{i\pi/2} \end{bmatrix}$

Table S3. Driving mechanisms and Raman tensors for the different vibrational modes.

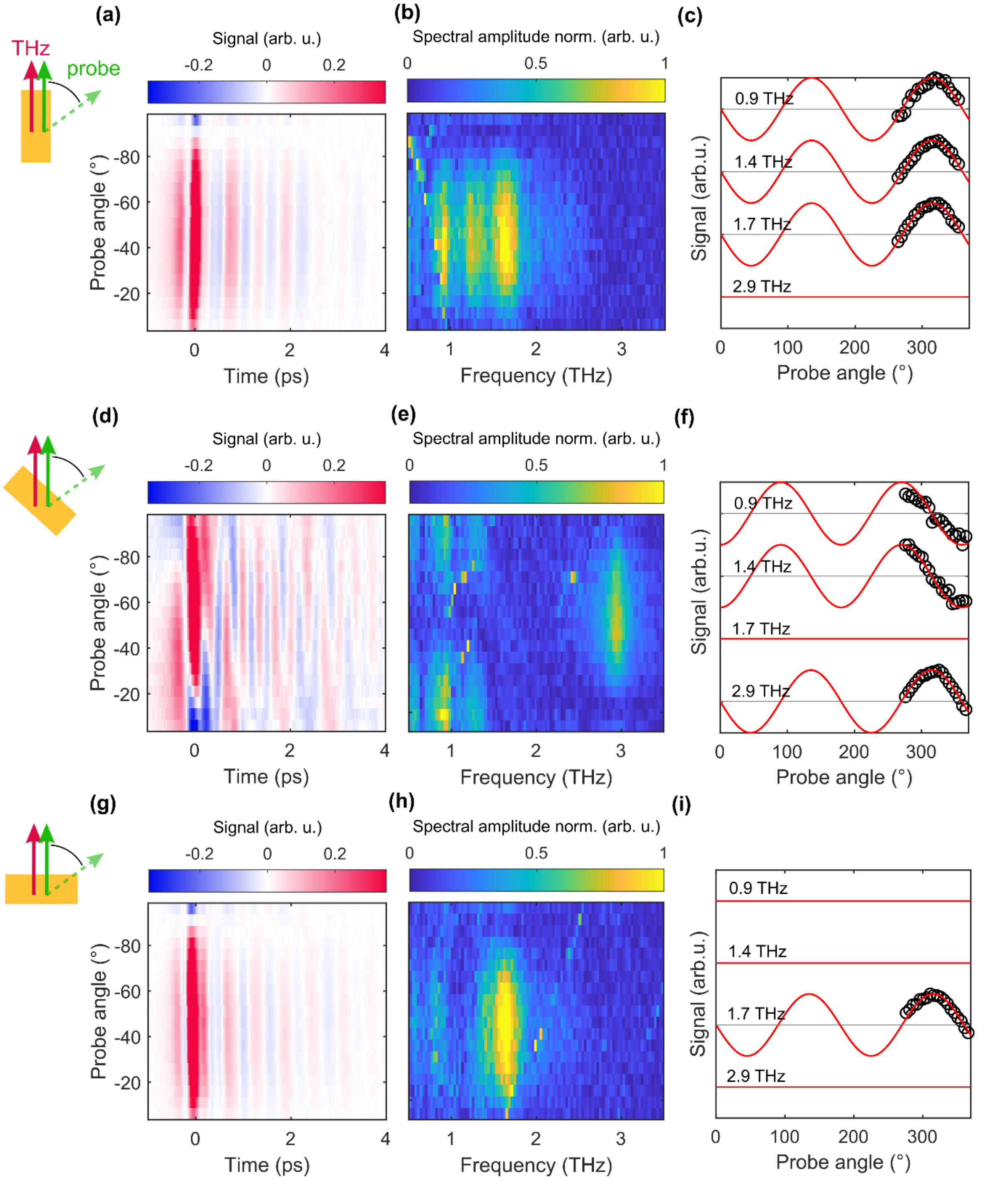

Figure S12. Probe polarization scans for flake long axis oriented parallel to the THz (**a)-c)**), at -45° (**d)-f)**) and perpendicular (**g)-i)**). Panels **a)**, **d)**, **g)**: transient birefringence signal as a function of probe polarization angle. Panels **b)**, **e)**, **h)**: signal Fourier transform amplitude. Panels **c)**, **f)**, **i)**: experimental (black dots) and theoretically calculated (red lines) signal amplitude as a function of probe polarization angle for the different Raman-active modes.

Section S12. Raman and IR driving mechanism simulations for the 0.9 and 1.4 THz modes

To confirm the assignment of the symmetries and driving mechanisms for the 0.9 THz and 1.4 THz mode, which show the complex multi-lobed transient birefringence pattern as a function

of the sample azimuthal rotation angle, we calculated the driving force F_{dr}, probing sensitivity S_{pr}, and transient birefringence signal using alternative forms of the mode's Raman tensors. We compared the results obtained using the tensors:

$$\mathbf{R}^{\alpha} = a\begin{bmatrix} 1 & \frac{4}{3}e^{i\pi/2} \\ \frac{4}{3}e^{i\pi/2} & -1 \end{bmatrix}, \qquad \mathbf{R}^{\gamma} = \begin{bmatrix} a & 0 \\ 0 & c \end{bmatrix}, \tag{S12}$$

with real $c \neq a$, $c>0$, $a>0$, and assumed either Raman, or IR driving as described in detail in Section S18. The comparison between the theoretical signals calculated using $\mathbf{R}^{\alpha}$ and $\mathbf{R}^{\gamma}$ shows that also this alternative Raman tensor including *xy* in-plane anisotropy cannot explain the observed angle dependence in a Raman driving – Raman probing picture. Figure S13 shows that only the combination of IR driving and probing via $\mathbf{R}^{\alpha}$ reproduces the experimental observations, in particular the signal sign changing as a function of ψ.

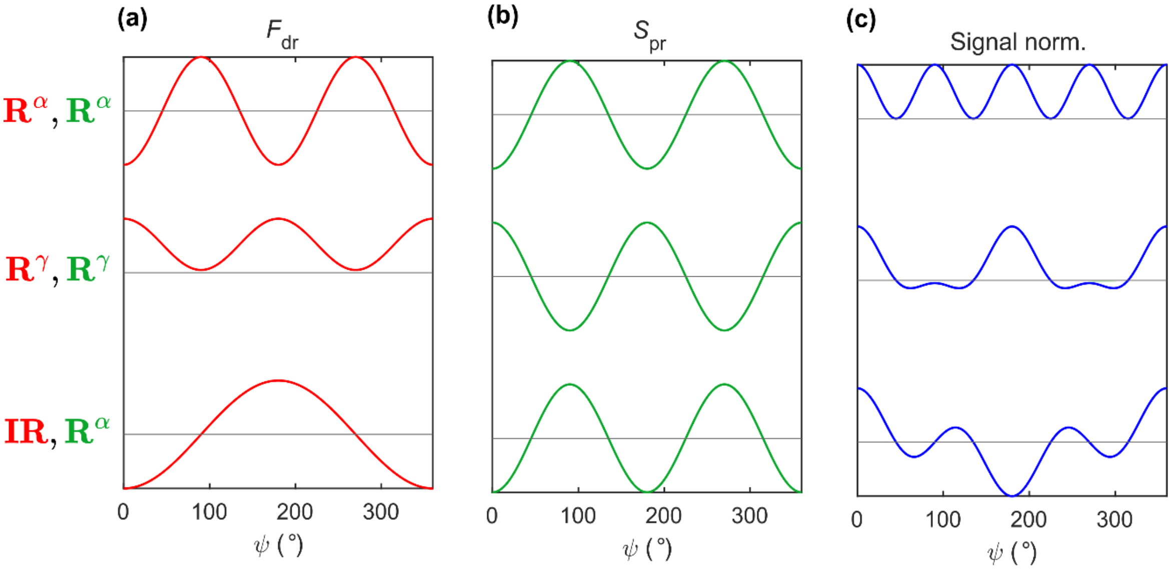

Figure S13. Calculated **a)** driving force, **b)** probing sensitivity, **c)** transient birefringence signal as a function of sample azimuthal angle for three different combinations of driving and probing mechanisms: Raman-type driving and probing described by $\mathbf{R}^{\alpha}$ (top)**,** Raman-type driving and probing described by $\mathbf{R}^{\gamma}$(middle) and IR driving and Raman-type probing described by $\mathbf{R}^{\alpha}$ (bottom). The black lines indicate zero of the calculated functions. The signal corresponds to the product of F_{dr} and S_{pr}. We use an unphysically high anisotropy ratio of the $\mathbf{R}^{\gamma}$ tensor elements c=20a to demonstrate the effect more clearly.

Section S13. Azimuthal angle scans for *n*=2,3

We measured transient birefringence signals for samples with *n*=2,3 inorganic layers as a function of the crystal azimuthal angle. Angle scans for *n*=1,2 and 3 are compared in **Figure S14 a-f**. For all the samples, the signal originating from the lowest-frequency modes shows a dependence on the field polarity, suggesting IR-type driving. This can be visualized by the

imaginary part of the Fourier transform **d-f**, as well as by comparing the inverse Fourier transforms after spectral filtering upon a 180° rotation, shown for the dominant low frequency modes in **Figure S14 j-l**. The balancing was not adjusted during the scans. While, as shown in section S8, the effect of this is mostly negligible for n=1, we did not perform such cross-checks for n=2,3, therefore the exact relative intensities measured in the angle scan may carry some error. The relative intensities at different angles ψ also carry an error due to sample inhomogeneities and imperfect centering of the rotation axis in the experimental setup.

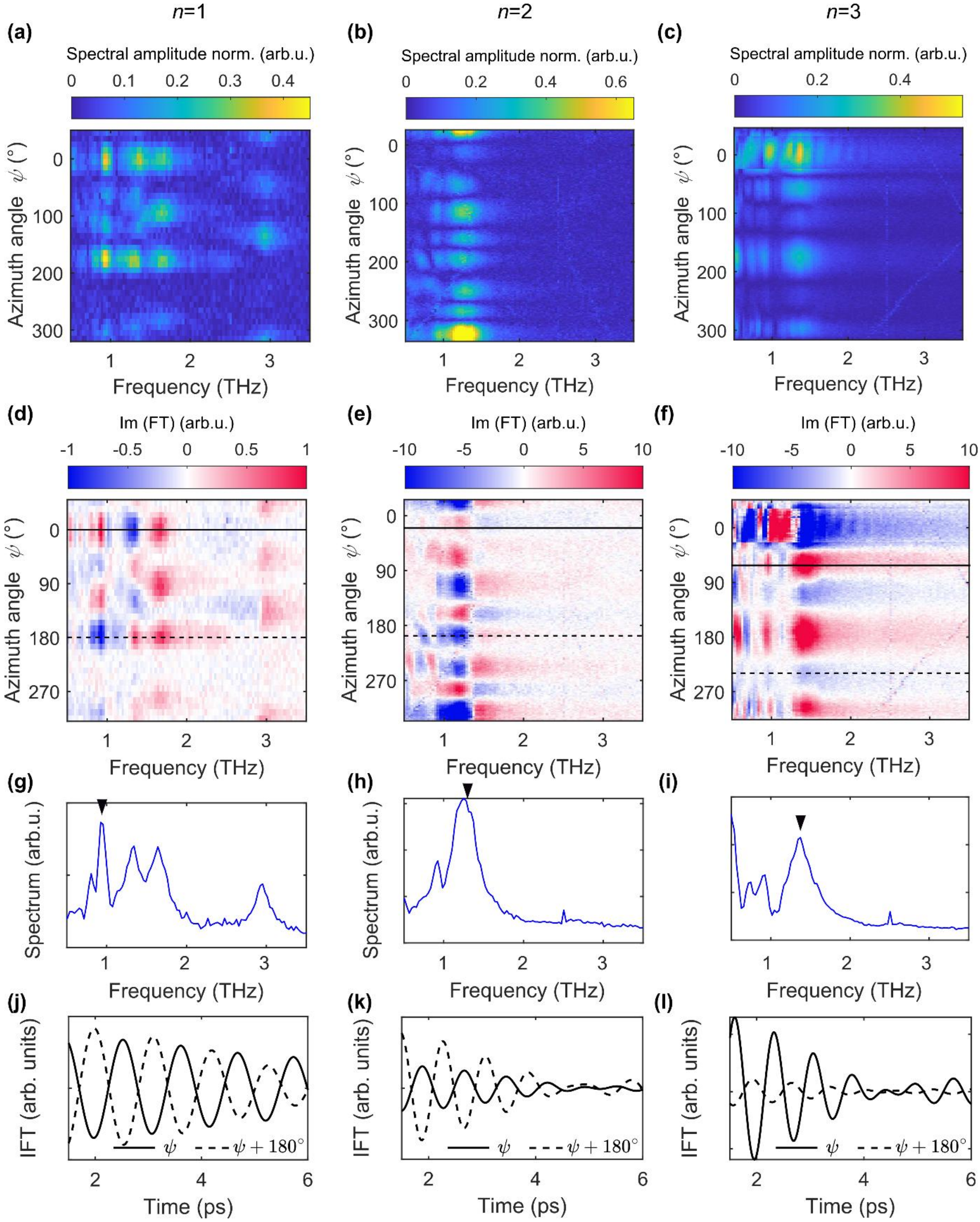

Figure S14. **a)-c)** spectral amplitude of the transient birefringence signal FT for n=1,2,3 samples as a function of azimuthal angle and **d)-f)** the corresponding imaginary part of the FTs. **g)-i)** Spectra averaged over 180°. **j)-l)** Inverse Fourier transforms calculated after spectral filtering around the dominating peaks marked with black triangles in panels g)-i), for two angles marked in panels d)-f) with the solid and dashed lines.

Section S14. Raman spectroscopy

We characterized the n=1 and n=2 using polarization-resolved spontaneous Raman scattering spectroscopy. The measurements were performed under ambient conditions, using a 647 nm excitation laser. **Figure S15** shows static Raman spectra measured on two different spots for each of the samples: with unpolarized detection for arbitrary excitation laser polarization (grey, the sum of the co- and cross-polarized spectra averaged over a full polarization scan (black), as well as the azimuthal angle-averaged transient birefringence spectrum squared (blue). The Raman spectra show good agreement between the different spots.
For n=1, the 0.9 THz mode, prominent in the transient birefringence spectrum, is negligible in the spontaneous Raman spectra. This can be due to its weak Raman activity, its dominance in the transient birefringence experiments being related to the very efficient linear direct driving. Relative differences in mode amplitude between the two experiments may be related to the driving force spectral profile, as discussed in Section S4. Certain modes appear absent in the transient birefringence spectrum (2.15 THz and 3.4 THz), which may be due to the balanced detection scheme being uniquely sensitive only to anisotropic modes.

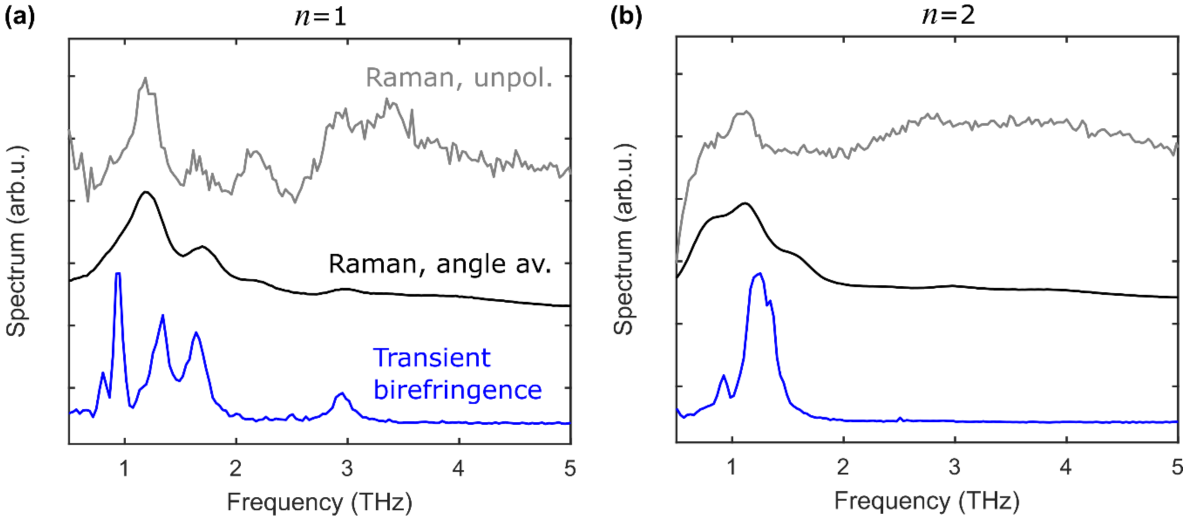

Figure S15. Comparison between spontaneous Raman scattering and transient birefringence measurements for **a)** n=1 and **b)** n=2. Grey: Raman spectrum for arbitrary excitation polarization and unpolarized detection. Black: sum of the Raman signal collected in co- and cross-polarized configuration, averaged over the sample azimuthal angles ψ: $\sum_\psi(I_\parallel(\psi)) + \sum_\psi(I_\perp(\psi))$. Blue: squared transient birefringence spectrum, averaged over ψ: $\sum_\psi|\mathrm{FT}(S(\psi))|^2$.

Figure S16 shows intensity maps of the Raman spectra measured in the co- and cross-polarization configuration as a function of the laser in-plane polarization angle for the two samples. The marked dominant modes show 2-fold and 4-fold symmetry, with maxima for laser polarization along the long flake edge direction, or at 45° to it.

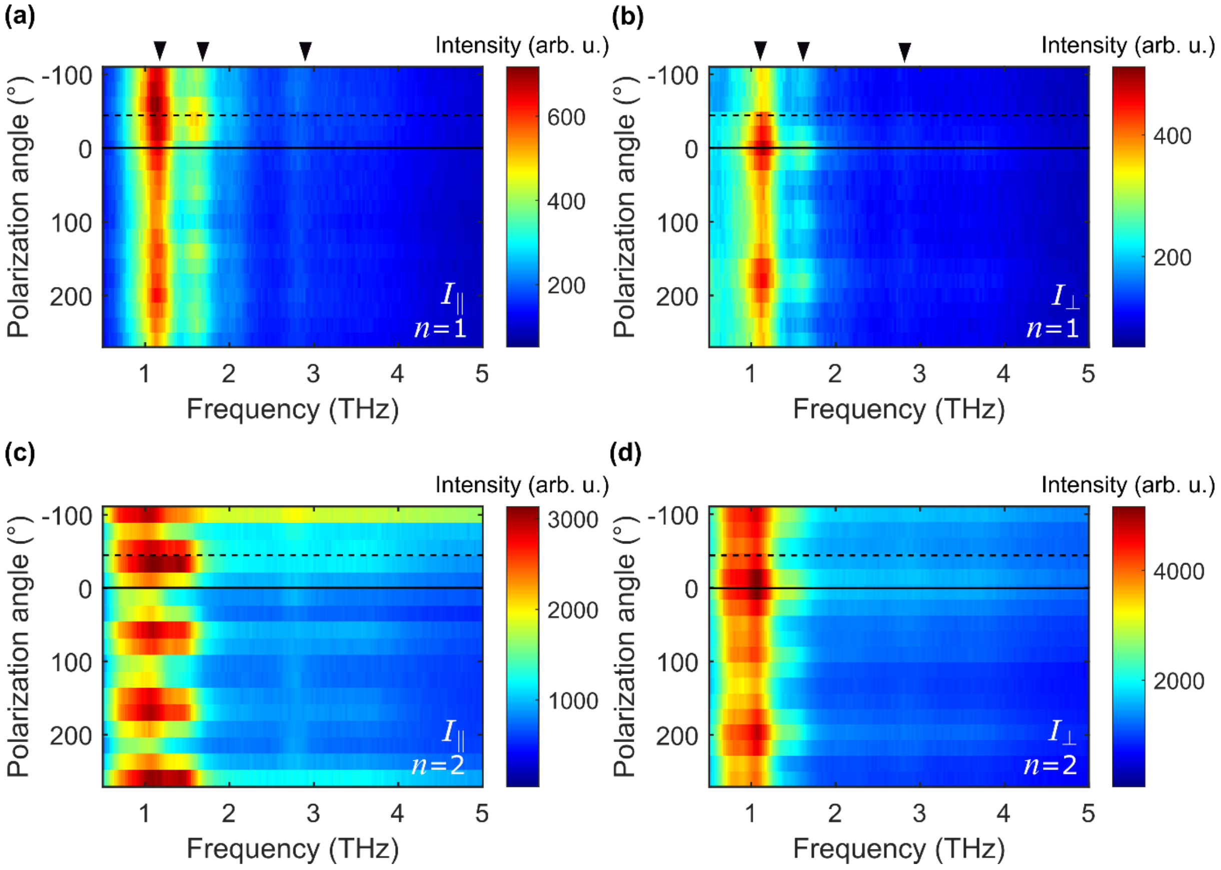

Figure S16. Polarized Raman spectra for n=1 **a)** and **b)**) and n=2 **c)** and **d)**) as a function of the excitation polarization angle. Maps in a) and c) show to co-polarized spectra, and b) and d) cross-polarized spectra. Dominant modes at ~1.2 THz, 1.7 THz and 2.9 THz are marked for n=1.The solid and dashed lines in all panels correspond to excitation polarization at 0° and 45° to the long crystal axis, respectively.

Figure S17 shows Raman spectra detected in both polarization configurations with the laser polarized at 0° and 45° to the long flake edge, corresponding to the lines in Figure S16, and squared transient birefringence spectra for the THz at 0° and 45° to the long flake edge.

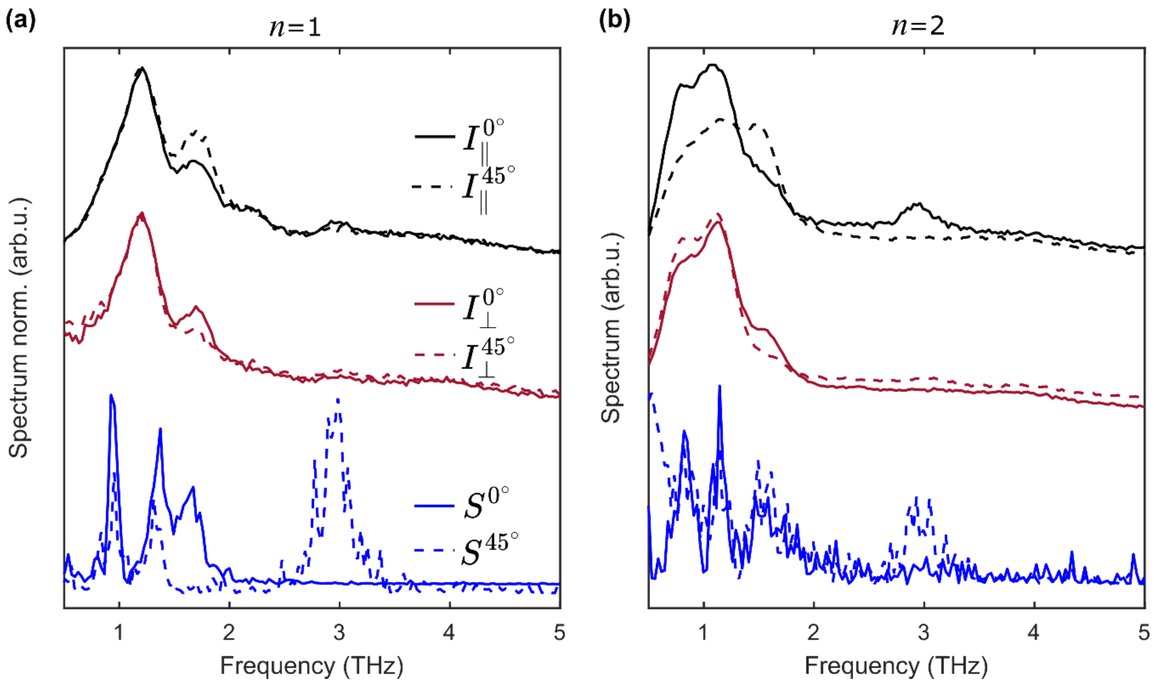

Figure S17. Co- (black) and cross-polarized (red) Raman spectra and squared transient birefringence spectra (blue) for *n*=1 **a)** and **c)**) and *n*=2 **b)** and **d)**). Solid lines show Raman spectra acquired at excitation polarization at 0° to the flake long edge, and transient birefringence measured for THz polarization parallel to the flake long axis. Dashed lines show spectra measured at the respective angles of 45° to the flake long edge.

Figure S18 a, b, f, g shows polar plots of the dominant 1.7 THz and 2.9 THz Raman peak experimental intensities as a function of the excitation polarization angles in the co- and cross-polarized configuration compared with theoretical calculations based on assigning the tensors of the modes as $\mathbf{R}^{\alpha}$ and $\mathbf{R}^{\beta}$, as given in the Main text and Table S3, respectively. The Raman peak intensity measured in those configurations is given by $I_{co} \propto |\hat{e}_i \mathbf{R} \hat{e}_i|^2$ and $I_{cross} \propto |\hat{e}_i^{\perp} \mathbf{R} \hat{e}_i|^2$, where $\hat{e}_i$ is a vector along the polarization direction of the incident laser beam and $\hat{e}_i^{\perp}$ perpendicular to it and $\mathbf{R}$ the mode Raman tensor. The tensor elements are obtained based on fitting the Raman intensity angle dependence up to a phase factor of $e^{i\pi/2}$. The final phase is assigned to obtain agreement with the transient birefringence experimental results.

Figure S18 c, d, i, h shows the azimuthal angle dependence of the driving force amplitude and probing sensitivity, as derived in **Section 17**. The probing sensitivity is calculated using $\mathbf{R}^{\alpha}$ and $\mathbf{R}^{\beta}$ for the two modes. We use the real part of the respective tensors to describe the driving process. **Figure S18 e, j** shows the corresponding experimentally measured and calculated TKE signal amplitude, as described in **Section S18**.

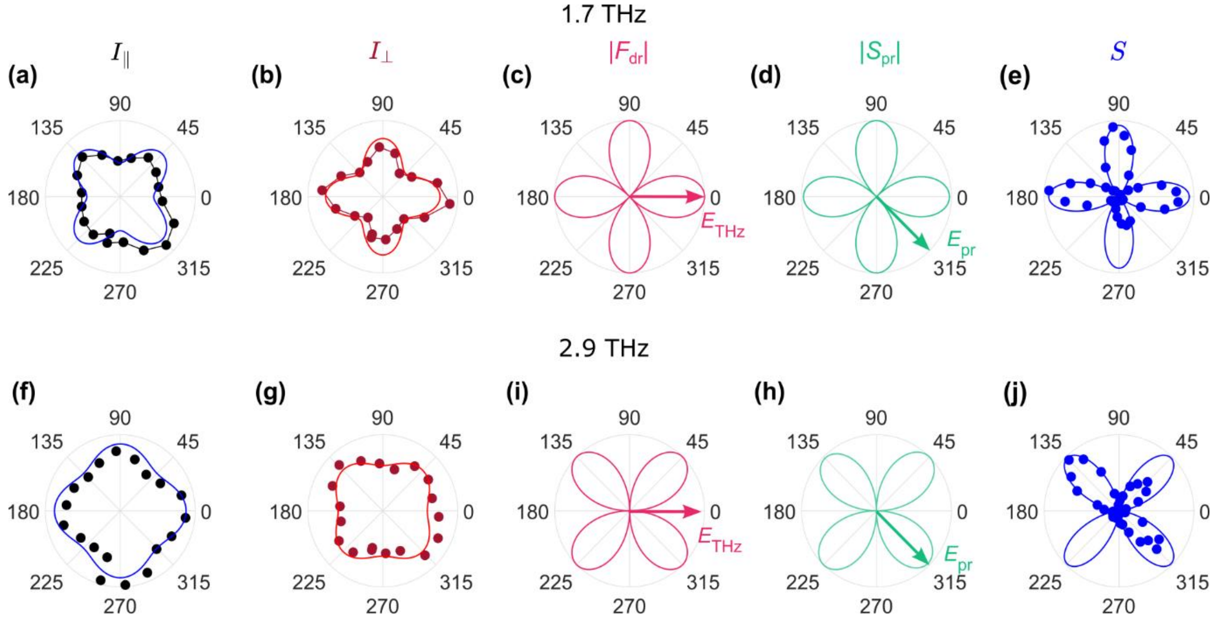

Figure S18. **a)-e)** 1.7 THz mode: Co-polarized ($I_{\parallel}$, black, **a)**) and cross-polarized ($I_{\perp}$, red, **b)**) Raman intensity, driving force amplitude **c)**, probing sensitivity **d)** and TKE signal amplitude (blue, **e)**). Points show experimental data and solid lines calculations based on the assigned Raman tensors. $\psi = 0^{\circ}$ corresponds to long flake edge and the arrows mark the THz pump and probe polarization directions. **f)-j)** 2.9 THz mode.

Section S15. Transient birefringence measurements on PEA halide salt crystals

Figure S19a shows the transient birefringence traces acquired at room temperature on PEAI and PEABr crystals, compared with the trace for the *n*=1 perovskite sample. For the ligand-halide crystals, thin samples (on BK7 glass substrates) and thicker crystals (**Figure S19d**) are compared. While clear oscillations are visible in both PEABr traces on the ps timescale, PEAI does not show distinguishable coherent phonon signatures. This may be related to much higher structural disorder, as can be seen in the optical images in **d**, as PEAI in contrast to PEABr does not form well oriented crystals on the substrate but rather disordered thin films. PEAI thick crystals also show an incoherent response after THz excitation, which does not fully decay in our measurement window of tens of ps, likely due to heating effects and slow heat dissipation in PEAI. The instantaneous response in the thin films shows an unipolar character, following E_{THz}^2, in PEABr and *n*=1 perovskite and a bipolar form in PEAI, reminiscent of earlier measurements in butylammonium ligands.[20] In thick crystals, the response may be strongly affected by anisotropic propagation effects[4] and is therefore not representative. The differences between THz-driven dynamics between PEAI, PEABr and perovskite crystals point to a strongly synergistic character of the response. Panel **b** shows the spectra of the oscillatory response in PEABr and $(PEA)_2PbI_4$ thin films. The clearly distinct peaks observed in both materials demonstrate, that the low-frequency rigid-body modes of the organic ligand molecules are highly sensitive to the local environment and the entire hybrid crystal structure. The vibrational response is strongly anisotropic for the highly ordered PEABr crystals, as shown in the azimuthal angle scan in panel **c**, which evidences the tendency of the PEA molecules to form highly ordered structures.

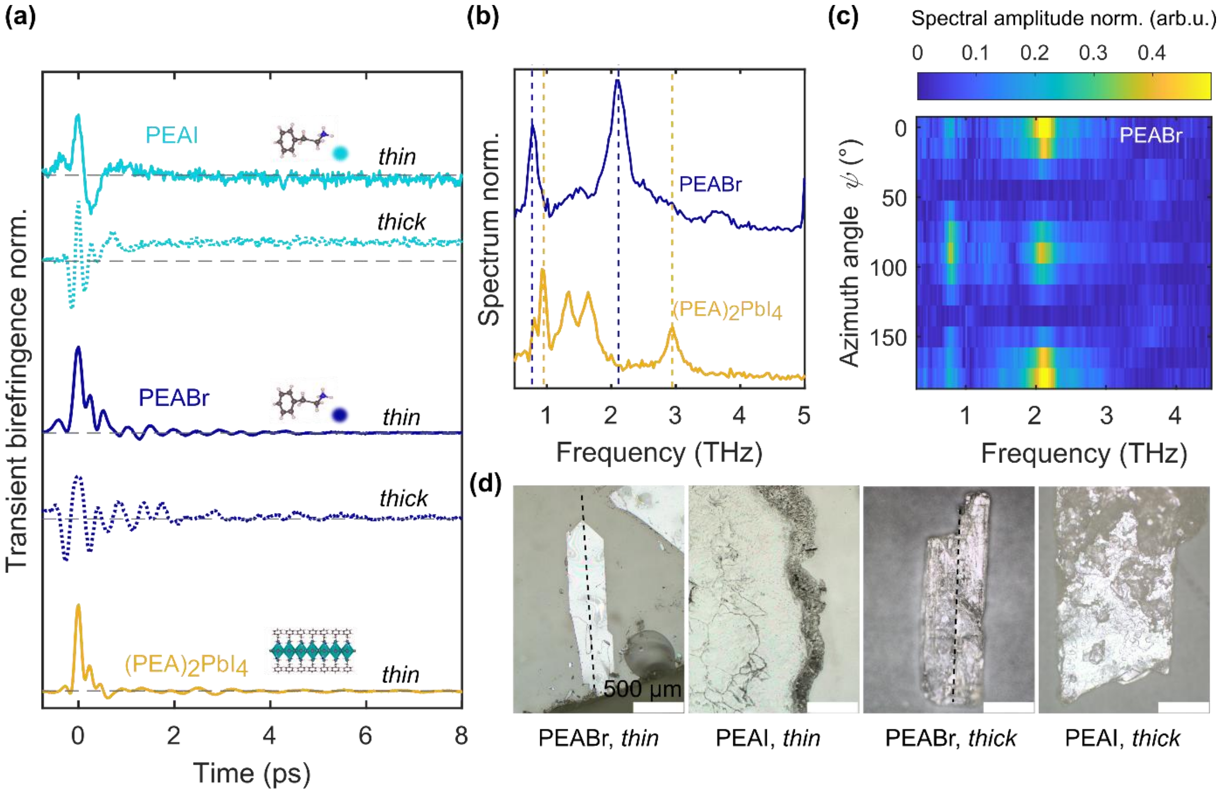

Figure S19. **a)** Normalized transient birefringence traces for thin and thick PEAI and PEABr crystals and the n=1 perovskite thin crystal sample. **b)** Normalized, azimuthal angle-averaged transient birefringence spectra for PEABr and n=1 thin crystals. **c)** Map of spectral amplitude for PEABr as a function of the sample azimuthal angle. **d)** Optical images of the measured ligand-halide crystals.

Section S16. THz transmission

We measured THz transmission through a thick, free-standing n=1 perovskite crystal for different sample azimuthal orientations via electro-optic sampling using a 100 µm ZnTe detection crystal. **Figure S20a** shows the Fourier transforms of the as-measured traces after clipping the signal in time domain to filter out pump and probe reflections. **Figure S20b** shows the relative transmittance at the respective angles, calculated by taking the ratios of the squared sample and reference signal spectral amplitudes, compared with an angle-averaged n=1 thin crystal transient birefringence spectrum. The relative transmittance is only meaningful for frequencies < 1.5 THz because of the signal to noise ratio at higher frequencies. A pronounced dip in the relative transmittance spectrum at around 0.9 THz corresponds to the dominant THz-induced transient birefringence peak, which we assigned to a simultaneously IR- and Raman-active mode.

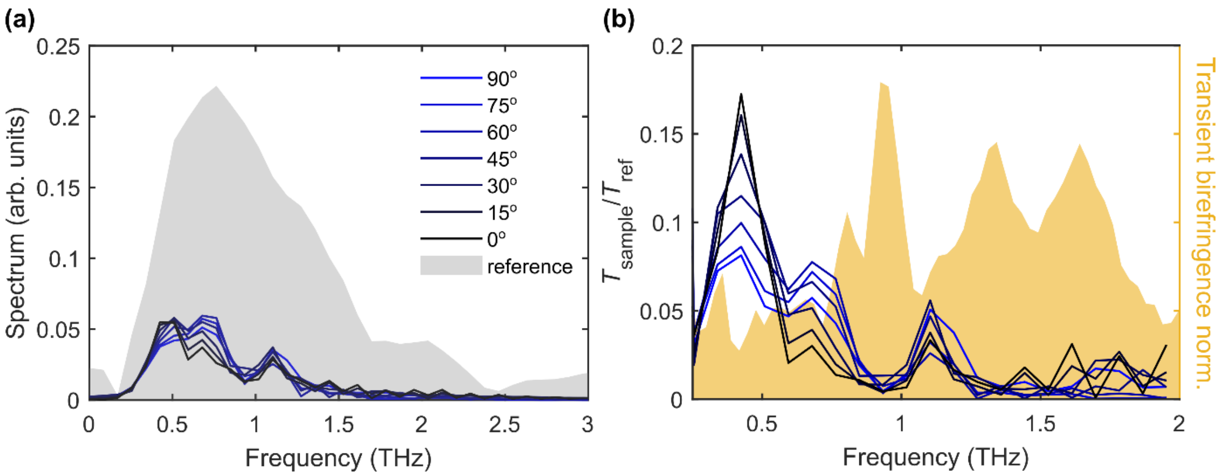

Figure S20. **a)** Spectral amplitude of electro-optic sampling signal, acquired without the sample (reference, grey area) and for the *n*=1 sample at different azimuthal angle orientations, where $\psi = 0^{\circ}$ corresponds to the long crystal axis parallel to the incident THz polarization. **b)** Relative transmittance $T_{\mathrm{sample}}/T_{\mathrm{ref}}$, compared with angle-averaged transient birefringence spectrum.

<u>**Section S17.** Theoretical description of coherent phonon driving and probing</u>

S17.1. Coherent phonon driving

In the discussion of our experimental results, we consider two types of coherent phonon generation mechanisms: linear and nonlinear photonic Raman-type driving.
Linear driving is possible for IR-active (polar) modes with resonance frequencies within the driving field spectrum. IR-active modes are associated with a change of the macroscopic polarization per unit cell with the mode normal coordinate, which means that the mode has nonzero effective charge. The *Born effective charge tensor* $\mathrm{Z}^{*}_{n,ij}$ contribution of the *n*-th ion is defined as:[21]

$$\mathrm{Z}^{*}_{n,ij} = \mathrm{V}_0 \frac{\partial \mathcal{P}_j}{\partial u_{n,i}}\bigg|_{E=0}, \qquad \text{(S13)}$$

where V_0 is the unit cell volume, $u_{n,i}$ the displacement of ion n along the spatial direction i and $\mathcal{P}_j$ the macroscopic polarization in the unit cell along the direction j for zero macroscopic electric field E. Modelling coherent phonons as driven, damped harmonic oscillators and assuming a harmonic lattice potential, in frequency domain we write the equation of motion for an IR-active mode with the normal mode coordinate $Q_{\mathrm{IR}}(\Omega)$ under external electric field $\boldsymbol{E}^{\mathrm{dr},1}(\omega_1)$ of the driving pulse as:[22]

$$(\Omega_{\mathrm{IR}}^2 - \Omega^2 - i\Gamma\Omega)Q_{\mathrm{IR}} = F_{\mathrm{dr}}^{\mathrm{IR}}(t) = \boldsymbol{\mathcal{Z}}^{*} \cdot \boldsymbol{E}^{\mathrm{dr},1}(\omega_1), \qquad \text{(S14)}$$

where Γ is the damping coefficient, Ω_{IR} the mode eigenfrequency and $\boldsymbol{\mathcal{Z}}^{*} = \sum_n Z_n^{*} u_n$ is the *mode effective charge vector*[22] coming from the summation over the contributions of all the ions in the unit cell. $F_{\mathrm{dr}}^{\mathrm{IR}}$ is the driving force for the IR-active mode. Phonon driving in this case occurs via a one-field interaction.

Modes for which $\boldsymbol{Z}^* = 0$ can be driven via a two-field, Raman-type interaction. For these modes, the linear susceptibility $\boldsymbol{\chi}^{(1)}(Q_\mathrm{R})$ changes as a function of the normal mode coordinate and can be expanded into a Taylor series for small perturbations around the equilibrium position $Q_\mathrm{R} = 0$:

$$\boldsymbol{\chi}^{(1)}(Q_\mathrm{R}) = \boldsymbol{\chi}_0^{(1)} + \left.\frac{\partial \boldsymbol{\chi}^{(1)}}{\partial Q_\mathrm{R}}\right|_{Q_\mathrm{R}=0} Q_\mathrm{R} + \cdots, \tag{S15}$$

where the derivative $\left.\frac{\partial \chi_{ij}{}^{(1)}}{\partial Q_\mathrm{R}}\right|_{Q_\mathrm{R}=0} \equiv \mathbf{R}_{ij}$ defines the *Raman susceptibility tensor*.[23] The Raman tensor element values are in general frequency dependent due to the dispersion of $\boldsymbol{\chi}^{(1)}$ and specific to a particular mode at frequency Ω_R: $\mathbf{R}^{\omega,\Omega_\mathrm{R}} = \left.\frac{\partial \chi^{(1)}(\omega)}{\partial Q_\mathrm{R}}\right|_{Q_\mathrm{R}=0}$. In the presence of a driving electric field component $\boldsymbol{E}^{\mathrm{dr},1}$ of the frequency ω_1, the induced nonequilibrium polarization $\boldsymbol{P}(Q_\mathrm{R}, \boldsymbol{E}^{\mathrm{dr},1})$ in the material is then given by:[23]

$$\boldsymbol{P}\left(Q_\mathrm{R}, \boldsymbol{E}^{\mathrm{dr},1}\right) = \varepsilon_0 \boldsymbol{\chi}^{(1)}(\omega_1, Q_\mathrm{R})\boldsymbol{E}^{\mathrm{dr},1} = \boldsymbol{P}_\mathrm{el} + \varepsilon_0 Q_\mathrm{R} \mathbf{R}^{\omega_1,\Omega_\mathrm{R}} \boldsymbol{E}^{\mathrm{dr},1}, \tag{S16}$$

with the first non-resonant term $\boldsymbol{P}_\mathrm{el} = \varepsilon_0 \boldsymbol{\chi}_0^{(1)} \boldsymbol{E}^{\mathrm{dr},1}$, corresponding to instantaneous electronic polarization, and the second term, modulated as a function of the Raman-active mode displacement. Here, the Raman susceptibility tensor $\mathbf{R}^{\omega_1,\Omega_\mathrm{R}}$ is defined for the driving electric field frequency ω_1, and the Raman phonon natural frequency Ω_R. The potential energy W of the nonequilibrium polarization under a second driving electric field component, $\boldsymbol{E}^{\mathrm{dr},2}$, is given by $W = -\boldsymbol{E}^{\mathrm{dr},2} \cdot \boldsymbol{P}\left(Q_\mathrm{R}, \boldsymbol{E}^{\mathrm{dr},1}\right) = \boldsymbol{E}^{\mathrm{dr},2} \cdot \boldsymbol{P}_\mathrm{el} + \varepsilon_0 Q_R \boldsymbol{E}^{\mathrm{dr},2} \cdot \mathbf{R}^{\omega_1,\Omega_\mathrm{R}} \boldsymbol{E}^{\mathrm{dr},1}$. The driving force for the collective dynamics of a Raman-active vibrational mode from this is as follows:

$$F_\mathrm{dr}^\mathrm{R} = -\frac{\partial W}{\partial Q_\mathrm{R}} = \varepsilon_0 \boldsymbol{E}^{\mathrm{dr},2} \mathbf{R}^{\omega_1,\Omega_\mathrm{R}} \boldsymbol{E}^{\mathrm{dr},1}. \tag{S17}$$

As can be seen from Equation (S17), two field interactions are necessary to drive a Raman-active mode. We assume that the Raman tensor $\mathbf{R}^{\omega_1,\Omega_\mathrm{R}}$ describing the coherent driving process has purely real elements. Under this assumption, using the real part of the Raman tensors obtained from fitting the spontaneous Raman results in the visible range as $\mathbf{R}^{\omega_1,\Omega_\mathrm{R}}$, we obtain very good agreement of our theoretical description with experimental results. In our case, $\boldsymbol{E}^{\mathrm{dr},1}$and $\boldsymbol{E}^{\mathrm{dr},2}$ are two THz electric field components at frequencies ω_1, ω_2 from within the bandwidth of the driving pulse. Considering energy conservation for the Raman process, the eigenfrequency of the coherently-driven Raman mode Ω_R has to be equal to $\Omega_\mathrm{R} = (\omega_1 + \omega_2)$ or $\Omega_\mathrm{R} = (\omega_1 - \omega_2)$ for a sum-frequency (analogous to two photon absorption[24]) or difference-frequency excitation process, respectively.[22,24] The equation of motion for the Raman-active mode then becomes:[22]

$$(\Omega_\mathrm{R}^2 - \Omega^2 - i\Gamma\Omega)Q_\mathrm{R} = F_\mathrm{dr}^\mathrm{R} = \varepsilon_0 \boldsymbol{E}^{\mathrm{dr},2} \mathbf{R}^{\omega_1,\Omega_\mathrm{R}} \boldsymbol{E}^{\mathrm{dr},1}. \tag{S18}$$

In principle, another excitation mechanism can lead to an observed quadratic dependence of the mode amplitude on the driving THz electric field, the so-called ionic driving mechanism. In this pathway, the THz electric field directly excites IR-active modes which subsequently anharmonically couple to drive a Raman-active mode.[25] Distinguishing between the Raman-type photonic and ionic mechanism is beyond the scope of this work and for simplicity we focus here on the photonic mechanism which, in contrast to the ionic mechanism, is universally possible.

S17.2. Coherent phonon detection

Coherent phonon probing in our detection scheme relies on the modulation of the linear susceptibility $\boldsymbol{\chi}^{(1)}(\omega_{\mathrm{pr}}, Q_{\mathrm{R}})$ at the frequency ω_{pr} of the probing field by the collective Raman-active phonon motion. We again Taylor-expand the susceptibility as a function of the vibrational coordinate Q_R:

$$\boldsymbol{\chi}^{(1)}\left(\omega_{\mathrm{pr}}, Q_{\mathrm{R}}\right) = \boldsymbol{\chi}_0^{(1)}(\omega_{\mathrm{pr}}) + \frac{\partial \boldsymbol{\chi}^{(1)}(\omega_{\mathrm{pr}})}{\partial Q_{\mathrm{R}}} Q_{\mathrm{R}} + \cdots. \tag{S19}$$

$\mathbf{R}^{\omega_{\mathrm{pr}},\Omega_{\mathrm{R}}} = \left.\frac{\partial \chi^{(1)}(\omega_{\mathrm{pr}})}{\partial Q_{\mathrm{R}}}\right|_{Q_{\mathrm{R}}=0}$ is the Raman susceptibility tensor, this time considered for ω_{pr}. The linear susceptibility is modulated by the oscillating phonon only if the mode is Raman-active, i.e. if the second term of Equation (S19) is nonzero. We are therefore only sensitive to Raman-active modes in our detection scheme. These can be purely Raman-active, or both IR- and Raman-active in an inversion symmetry-broken material, such as the 0.9 THz and 1.4 THz modes considered in our work.

The nonequilibrium polarization $\boldsymbol{P}_{\mathrm{pr}}(Q_{\mathrm{R}}, \boldsymbol{E}^{\mathrm{pr}})$ induced in the material in the presence of the probe field, written as a function of the coherent phonon vibrational coordinate is therefore:

$$\boldsymbol{P}_{\mathrm{pr}}(Q_{\mathrm{R}}, \boldsymbol{E}^{\mathrm{pr}}) = \varepsilon_0 \boldsymbol{\chi}^{(1)}\left(\omega_{\mathrm{pr}}, Q_{\mathrm{R}}\right) \cdot \boldsymbol{E}^{\mathrm{pr}} = \boldsymbol{\chi}_0^{(1)}(\omega_{\mathrm{pr}}) \boldsymbol{E}^{\mathrm{pr}} + \varepsilon_0 Q_{\mathrm{R}} \mathbf{R}^{\omega_{\mathrm{pr}},\Omega_{\mathrm{R}}} \boldsymbol{E}^{\mathrm{pr}} \tag{S20}$$

The first term of Equation (S20) includes the linear, as well as pump field-induced nonlinear polarization, related to the nonresonant electronic response. In the second term of the right-hand side of Equation (S20), the phonon amplitude Q_{R} is a function of the driving field and corresponds to an effective nonlinear polarization. The solutions for the coherent phonon equations of motion (S14) and (S18) in the case of linear and Raman-type driving give us, respectively:

$$Q_{\mathrm{IR}}(\omega = \omega_1) = \frac{F_{\mathrm{dr}}^{\mathrm{IR}}(\omega)}{\Omega_{\mathrm{IR}}^2 - \omega^2 - i\Gamma\omega} = \frac{\boldsymbol{Z}^* \cdot \boldsymbol{E}^{\mathrm{dr},1}(\omega_1)}{\Omega_{\mathrm{IR}}^2 - \omega^2 - i\Gamma\omega}, \tag{S21}$$

$$Q_{\mathrm{R}}(\omega = \omega_1 \pm \omega_2) = \frac{F_{\mathrm{dr}}^{\mathrm{R}}(\omega)}{\Omega_{\mathrm{R}}^2 - \omega^2 - i\Gamma\omega} = \frac{\varepsilon_0 \boldsymbol{E}^{\mathrm{dr},2}(\omega_2) \cdot \mathbf{R}^{\omega_1,\Omega_{\mathrm{R}}} \boldsymbol{E}^{\mathrm{dr},1}(\omega_1)}{\Omega_{\mathrm{R}}^2 - \omega^2 - i\Gamma\omega}. \tag{S22}$$

From Equations (S20), (S21) and (S22) we obtain the expressions for the *phonon-modulated component of the nonlinear polarization* for the case of IR-driving and Raman driving, $\boldsymbol{P}_{\mathrm{pr}}^{(2)}(\omega,\omega_{\mathrm{pr}})$ and $\boldsymbol{P}_{\mathrm{pr}}^{(3)}(\omega_1,\omega_2,\omega_{\mathrm{pr}})$, respectively:

$$\boldsymbol{P}_{\mathrm{pr}}^{(2)}(\omega,\omega_{\mathrm{pr}}) \propto (\boldsymbol{\mathcal{Z}}^{*}\cdot\boldsymbol{E}^{\mathrm{dr},1}(\omega))(\mathbf{R}^{\omega_{\mathrm{pr}},\Omega_{\mathrm{IR,R}}}\,\boldsymbol{E}^{\mathrm{pr}}), \tag{S23}$$

$$\boldsymbol{P}_{\mathrm{pr}}^{(3)}(\omega_1,\omega_2,\omega_{\mathrm{pr}}) \propto (\varepsilon_0\boldsymbol{E}^{\mathrm{dr},2}(\omega_2)\cdot\mathbf{R}^{\omega_1,\Omega_{\mathrm{R}}}\boldsymbol{E}^{\mathrm{dr},1}(\omega_1))(\mathbf{R}^{\omega_{\mathrm{pr}},\Omega_{\mathrm{IR,R}}}\,\boldsymbol{E}^{\mathrm{pr}}). \tag{S24}$$

$\Omega_{\mathrm{IR,R}}$ is the frequency of a mode which is simultaneously IR and Raman-active, enabling both linear driving and Raman-type probing. Ω_{R} is the frequency of a Raman-driven mode. The polarization is second-order $\boldsymbol{P}_{\mathrm{pr}}^{(2)}(\omega,\omega_{\mathrm{pr}})$ in the case of linear driving of IR-and-Raman-active modes and third-order $\boldsymbol{P}_{\mathrm{pr}}^{(3)}(\omega_1,\omega_2,\omega_{\mathrm{pr}})$ for Raman-type driving. In the wave-mixing picture, we can introduce the effective nonlinear susceptibility tensors $\boldsymbol{\chi}_{\mathrm{eff}}^{(2)}$ and $\boldsymbol{\chi}_{\mathrm{eff}}^{(3)}$:

$$\boldsymbol{P}_{\mathrm{pr},i}^{(2)} = \boldsymbol{\chi}_{\mathrm{eff},ijk}^{(2)}\boldsymbol{E}_j^{\mathrm{pr}}\boldsymbol{E}_k^{\mathrm{dr},1} \propto \mathbf{R}_{ij}^{\omega_{\mathrm{pr}},\Omega_{\mathrm{IR,R}}}\boldsymbol{E}_j^{\mathrm{pr}}\boldsymbol{\mathcal{Z}}_k^{*}\boldsymbol{E}_k^{\mathrm{dr},1}, \tag{S25}$$

$$\boldsymbol{P}_{\mathrm{pr},i}^{(3)} = \boldsymbol{\chi}_{\mathrm{eff},ijkl}^{(3)}\boldsymbol{E}_j^{\mathrm{pr}}\boldsymbol{E}_k^{\mathrm{dr},1}\boldsymbol{E}_l^{\mathrm{dr},2} \propto \mathbf{R}_{ij}^{\omega_{\mathrm{pr}},\Omega_{\mathrm{R}}}\mathbf{R}_{kl}^{\omega_1,\Omega_{\mathrm{R}}}\boldsymbol{E}_j^{\mathrm{pr}}\boldsymbol{E}_k^{\mathrm{dr},1}\boldsymbol{E}_l^{\mathrm{dr},2}, \tag{S26}$$

where the repeating indices indicate summation.

The effective $\boldsymbol{\chi}_{\mathrm{eff}}^{(3)}$ is constructed as a product of two standard Raman susceptibility tensors, provided the absence of absorption (transparency at the probe frequency).[26] The effective $\boldsymbol{\chi}_{\mathrm{eff}}^{(2)}$ is analogously constructed from the product of the IR dipole and the Raman susceptibility tensor.[27] The nonlinear polarization is the source of signal fields $\boldsymbol{E}^{\mathrm{sig}}$ at frequencies shifted from the probe frequency by the phonon frequency Ω: $(\omega_{\mathrm{pr}} \pm \Omega)$. Our experiment is equivalent to heterodyne detection of signal fields generated in coherent anti-Stokes Raman scattering (CARS) and coherent Stokes Raman scattering (CSRS) processes,[28,29] with the transmitted probe field acting as the local oscillator in the heterodyne detection scheme.

S17.3. Signal field detection in balanced detection scheme

In our experiment, the signal fields co-propagate together with the remaining transmitted field $\boldsymbol{E}^{\mathrm{pr,tr}}$ towards the detection setup. Because $\boldsymbol{E}^{\mathrm{sig}}$ frequencies (for both the Stokes, as well as the anti-Stokes probing pathway, as described in the Main text and detailed in Fig. S24) lie within the spectral bandwidth of ~ 25 THz of the fs probe pulse, we can consider the superposition of the emitted signal and transmitted probe field as an effective change of the probe beam polarization state (transient birefringence picture[30]). The balanced detection scheme consists of a quarter- (QWP) and half-wave plate (HWP). During balancing, the HWP is first adjusted so that the static transmitted probe field amplitudes along the x and y directions are equal and the QWP is afterwards added and then adjusted to compensate for any static ellipticity. In this way, we afterwards convert the THz-induced elliptical polarization of the transmitted probe

beam to a rotation of its linear polarization. The sum of the signal field and probe field transmitted through the sample ($\boldsymbol{E}^{\text{sig}} + \boldsymbol{E}^{\text{pr,tr}}$) passes through a Wollaston prism which projects the field components polarized along the x and y directions to two separate photodiodes. We use ~~and~~ an incident probe polarization angle such that in the balanced condition in the absence of THz modulation, the transmitted probe field is along the $(-x, y)$ direction after the two waveplates.

The detected transient birefringence signal S is proportional to the intensity difference on the two diodes $S \propto (\boldsymbol{E}^{\text{sig}} + \boldsymbol{E}^{\text{pr,tr}})_x^2 - (\boldsymbol{E}^{\text{sig}} + \boldsymbol{E}^{\text{pr,tr}})_y^2$. In the balanced case $E_x^{\text{pr,tr}} = E_y^{\text{pr,tr}}$ and assuming the quadratic terms $(E_x^{\text{sig}})^2$and $(E_x^{\text{sig}})^2$ are negligibly small, $S \propto (E_x^{\text{sig}} - E_y^{\text{sig}})$.

As seen from the above expression, the signal S scales linearly with the amplitude of the nonlinear signal field. The phonon-modulated nonequilibrium polarization, which is the source of the signal field, in turn scales linearly with the coherent phonon amplitude (Equation S20). In the time domain the signal $S(t)$ therefore directly visualizes the instantaneous periodic displacement of the lattice mode $Q(t)$.

Section S18. Numerical simulation of the signal angle dependence

In our numerical calculation, we use the Jones formalism to implement the probe and pump electric field polarization vectors and optical elements. We use electric field vectors normalized to unity, and define the matrix describing the effect of a QWP at the angle corresponding to our lab geometry as (for simplicity here neglecting adjustments of the optics to compensate for static birefringence):

$$\mathbf{M}_{\text{QWP}}^{45^\circ} = \frac{1}{\sqrt{2}}\begin{bmatrix} 1 & i \\ i & 1 \end{bmatrix}. \tag{S29}$$

Similarly, we implement the HWP matrix:

$$\mathbf{M}_{\text{HWP}}^{45^\circ} = \begin{bmatrix} 0 & -1 \\ -1 & 0 \end{bmatrix}, \tag{S30}$$

and on describing the Wollaston prism:

$$\mathbf{M}_{\text{WP}}^{0^\circ} = \begin{bmatrix} 1 & 0 \\ 0 & -1 \end{bmatrix}. \tag{S31}$$

To simulate the azimuthal angle dependence experiment, we assume incident THz and probe fields propagating along z, with polarization identical to our experimental conditions:

$$\boldsymbol{E}^{\text{THz}} = \begin{bmatrix} 0 \\ 1 \end{bmatrix}, \qquad \boldsymbol{E}^{\text{pr}} = \frac{1}{\sqrt{2}}\begin{bmatrix} -1 \\ 1 \end{bmatrix} \tag{S32}$$

We define the Raman tensors for the different vibrational modes:

$$\mathbf{R} = \begin{bmatrix} a & b \\ b & c \end{bmatrix} \tag{S33}$$

under the assumption that $(|a|, |b|, |c| \ll 1)$, consistent with the fact that Raman scattering is generally a weak process and only considering the xy plane. We calculate the Raman-type driving force $F^{\text{dr}}(\psi)$ for every sample rotation angle ψ based on the rotated Raman tensor $\mathbf{R}^{\text{dr}}(\psi)$:

$$\mathbf{R}(\psi) = \boldsymbol{\mathcal{R}}(-\psi)\mathbf{R}^{\text{dr}}(\psi = 0)\boldsymbol{\mathcal{R}}(\psi) \tag{S34}$$

where $\boldsymbol{\mathcal{R}}(\psi)$ is the rotation matrix about the z axis by an angle ψ (assuming $\psi = 0$ for the crystal axis along the y direction):

$$\boldsymbol{\mathcal{R}}(\psi) = \begin{bmatrix} \cos(\psi) & \sin(\psi) \\ -\sin(\psi) & \cos(\psi) \end{bmatrix} \quad \text{(S35)}$$

The driving force for a given mode is then calculated as:

$$F^{\mathrm{dr}}(\psi) = \boldsymbol{E}^{\mathrm{THz}} \mathbf{R}^{\mathrm{dr}}(\psi) \boldsymbol{E}^{\mathrm{THz}} \quad \text{(S36)}$$

We calculate the signal field emitted by the nonlinear polarization $\boldsymbol{E}_{\mathrm{sig}}^{(3)}(\psi)$ as:

$$\boldsymbol{E}_{\mathrm{sig}}^{(3)}(\psi) = e^{-i\pi/2} F^{\mathrm{dr}}(\psi) \quad \text{(S37)}$$

The emitted signal field is phase-shifted by $-\pi/2$ relative to E_{probe}. This follows from the one-dimensional inhomogeneous wave equation, considering propagation along z, where the field emitted by the nonlinear polarization P_{NL} is given by:[31]

$$\frac{d\boldsymbol{E}_{\mathrm{sig}}(\omega_{\mathrm{pr}} \pm \Omega; z)}{dz} \propto iP_{\mathrm{NL}}(z), \quad \text{(S38)}$$

meaning that the signal field has a $-\pi/2$ phase shift relative to E_{pr}:

$$\boldsymbol{E}_{\mathrm{sig}} \propto e^{-\frac{i\pi}{2}} F^{\mathrm{dr}}(\psi)\, \mathbf{R}^{\mathrm{pr}} \boldsymbol{E}^{\mathrm{pr}} \quad \text{(S39)}$$

$\mathbf{R}^{\mathrm{pr}}$ is the Raman tensor describing the probing process. We approximate the total field after the sample as the sum of the incident probe field and the emitted signal field $\boldsymbol{E}^{\mathrm{tot}}(\psi)$=($\boldsymbol{E}^{\mathrm{pr}} + \boldsymbol{E}_{\mathrm{sig}}^{(3)}(\psi)$), which after passing through the balancing optics becomes:

$$\boldsymbol{E}^{\mathrm{det}}(\psi) = \mathbf{M}_{\mathrm{WP}}^{0^\circ} \mathbf{M}_{\mathrm{HWP}}^{45^\circ} \mathbf{M}_{\mathrm{QWP}}^{45^\circ} \boldsymbol{E}^{\mathrm{tot}}(\psi) \quad \text{(S40)}$$

The calculated intensity of the x and y polarization components on the photodiodes after the Wollaston prism are $I_x = \boldsymbol{E}_x^{\mathrm{det}} \cdot (\boldsymbol{E}_x^{\mathrm{det}})^*$ and $I_y = \boldsymbol{E}_y^{\mathrm{det}} \cdot (\boldsymbol{E}_y^{\mathrm{det}})^*$ and we therefore compute the normalized signal finally as:

$$S(\psi) = (I_x - I_y) \quad \text{(S41)}$$

We note that in our calculations the field vectors are normalized and the absolute values of the Raman tensor elements are arbitrary (while fulfilling the condition $\ll 1$). Therefore, we always obtain only the relative sign and magnitude of the driving force, signal field and measured transient birefringence signal as a function of the rotation angle ψ for a particular mode. While

this information is sufficient for our symmetry analysis, we cannot conclude on the differences in absolute phonon amplitudes or signal magnitudes between the different modes.

Section S19. Sample quality and stability

Figure S21 a shows the photoluminescence spectra of $(PEA)_2PbI_4$ *n*=1, 2 AVCC-grown thin crystal samples (backscattering geometry, 405 nm excitation, ambient conditions). The narrow strong excitonic peaks at 527 nm and 577 nm and absence of significant low-energy defect band are signatures of low defect densities and a high sample quality. X-ray diffraction data for AVCC samples prepared using an identical procedure have been previously published in Ref.[2] (*n*=1) and [32] (*n*=1, 2), confirming phase purity and high crystalline quality. **Figure S21 b** shows the magnitude of the instantaneous electronic response in the transient birefringence signal for *n*=1 as a function of the azimuthal angle for a full 360° scan starting from $\psi = -45^\circ$. **Figure S21 c** compares the signals measured for $\psi = -45^\circ$ at the beginning and end of the azimuthal angle scan for *n*=1, which was acquired under continuous probe pulse (800 nm) illumination over the period of around 6 hours. No significant changes of the signal are visible.

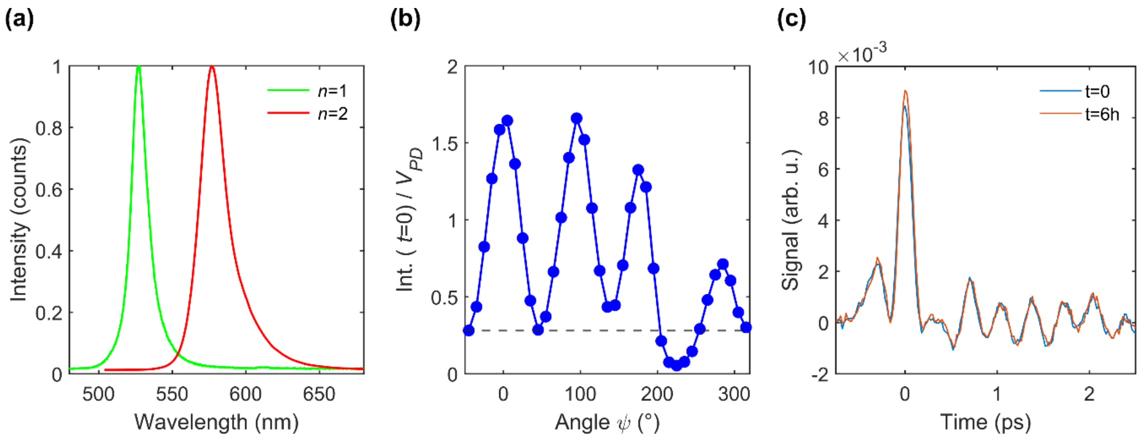

Figure S21. **a)** Photoluminescence spectra of the $(PEA)_2PbI_4$ *n*=1 and *n*=2 AVCC samples at room temperature. **b)** Instantaneous electronic response in the transient birefringence signal as a function of the azimuthal rotation angle for *n*=1. **c)** Signals measured for $\psi = -45^\circ$ at the beginning (*t*=0) and end ($t \approx$ 6h) of the azimuthal angle scan in b).

Figure S22 a shows the micro-photoluminescence spectra of samples synthesized using the slow-cooling method, confirming phase purity, and **Figure S22 b** the corresponding X-ray diffraction (XRD) data. For the XRD analysis, PbI_6 sheets were considered to be oriented in-plane along the surface. The lattice distances corresponding to the first diffraction peaks were calculated to be 1.64 nm, 2.236 nm, and 2.865 nm for the n = 1, 2, and 3 samples, respectively. The stepwise increase of approximately 0.6 nm between n = 1, 2, and 3 corresponds to the thickness of a single PbI_6 sheet, which provides a strong proof for the accurate crystal structure determination.

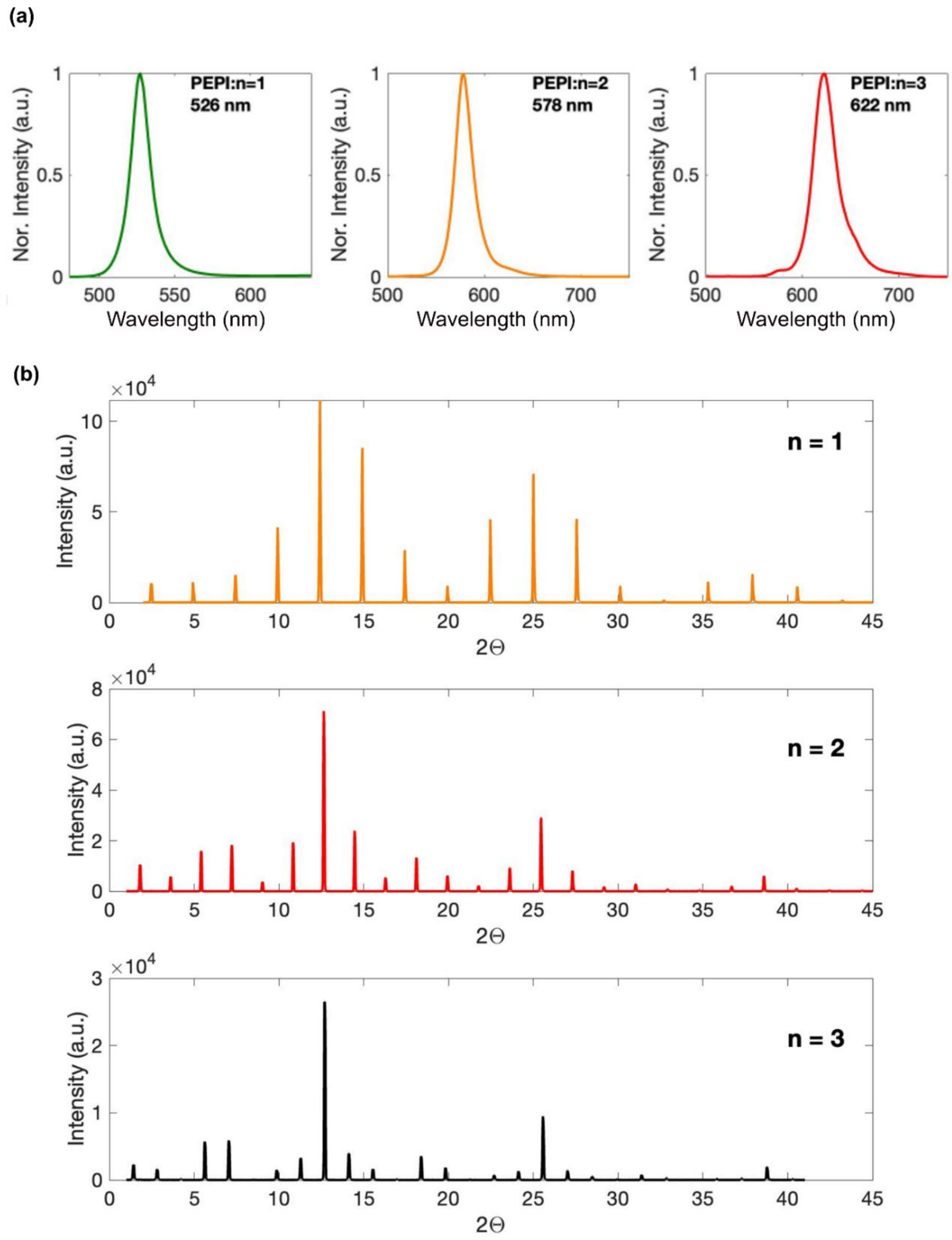

Figure S22 a) Micro-photoluminescence spectra of slow-cooling samples n=1,2,3 and **b)** the corresponding XRD patterns.

<u>**Section S20.** Second Harmonic Generation (SHG) and Multi-Photon Photoluminescence</u>

To verify the presence of SHG, we conducted two spectroscopic experiments on *n*=1 samples: First, we acquired $(PEA)_2PbI_4$ emission spectra in the visible range in transmission geometry under 800 nm excitation (using a long-pass filter to reject the remaining excitation beam). **Figure S23a** shows spectra measured in transmission geometry under 800 nm excitation, demonstrating strong multi-photon photoluminescence signals as well as the absence of

measurable SHG signal at 400 nm, likely due to strong reabsorption.[33] **Figure S23b** depicts the scaling of photoluminescence intensity with 800 nm excitation power, which, depending on the azimuthal angle follows a dependence characteristic of 2-photon ($\psi = 45^{\circ}$) or 3-photon ($\psi = 0^{\circ}$) luminescence. **Figure S23c** shows the dependence of the multi-photon luminescence intensity on the azimuthal angle.

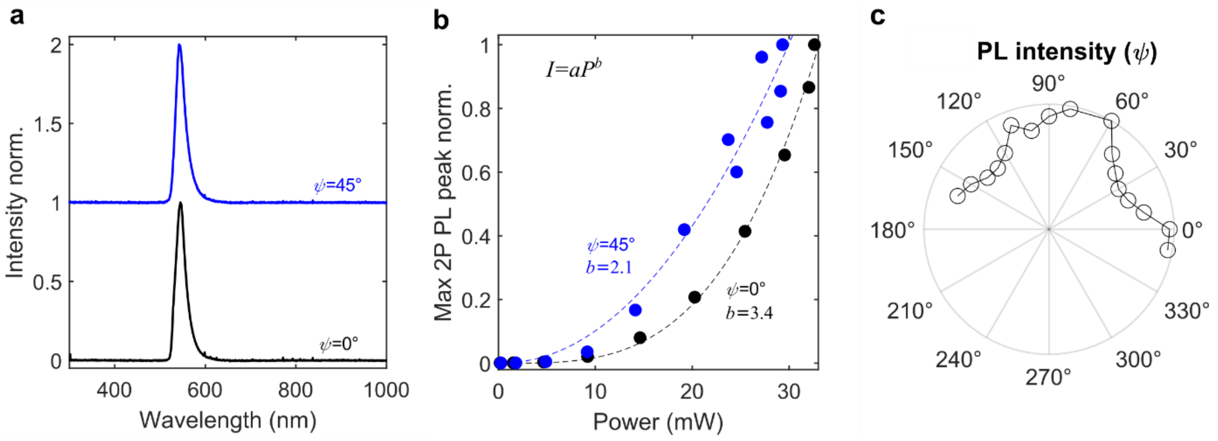

Figure S23. **a)** Multi-photon photoluminescence signal measured for two different sample azimuthal orientations. The 800 nm excitation was polarized along $\psi = 0^{\circ}$. **b)** Scaling of the intensity of the photoluminescence peak with excitation power and power law fits **c)** Photoluminescence intensity as a function of sample azimuthal angle.

Secondly, we tried to observe nonlinear sum- and difference-frequency 2nd order signals in a nonlinear time-domain microscopy setup as described in ref.[34,35] using below-bandgap input beams in the IR (3.45 μm , i.e. 2900 cm^{-1} / 87 THz/0.36 eV) and VIS (690 nm). **Figure S24** shows images acquired using below-band gap excitation in the IR (3.45 μm). Strikingly, the intensity of multi-photon photoluminescence observed with only IR excitation ($E_{g,optical} \approx 2.37$ eV $\approx 6.6 \cdot h\upsilon_{IR}$, where $E_{g,optical}$ is the optical bandgap of $(PEA)_2PbI_4$ at room temperature and υ_{IR} the IR photon frequency) is more than two orders of magnitude higher than that of a typical 2nd nonlinear order signal (mixing IR and visible excitation) measured on reference samples (e.g. bulk quartz) under comparable conditions. Such strong luminescence background precludes the observation of sum- or difference-frequency 2nd order signals and reliable conclusions on their presence or absence.

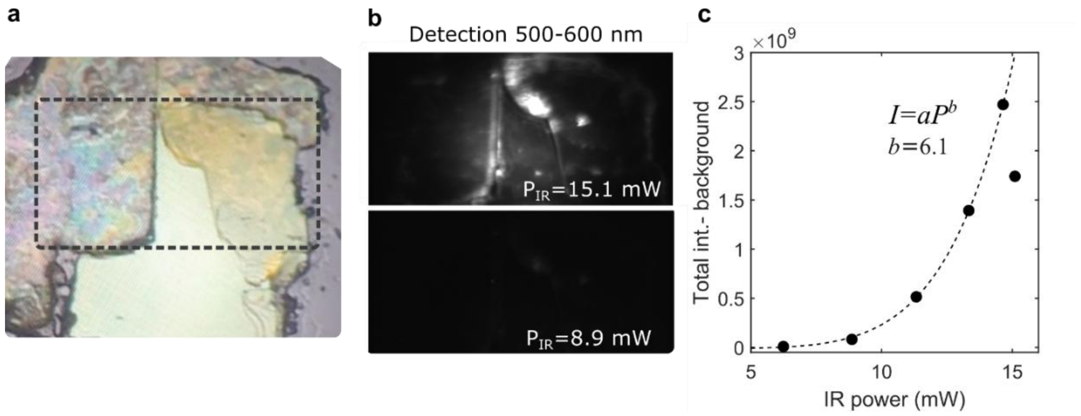

Figure S24. **a)** Optical microscope image of exfoliated $(PEA)_2PbI_4$ flake with the marked region corresponding to the images shown in panel b) **b)** Nonlinear microscopy imaging after spectral filtering in the 500-600 nm range under different IR excitation power P_{IR} **c)** Scaling of the detected intensity integrated over the entire image with P_{IR}. The dashed line shows a power law fit.

Section S21. Wave mixing energy level (WMEL) and Feynman diagrams

Figure S25 shows the WMEL and double-sided Feynman diagrams for the nonlinear processes discussed: resonant IR, Raman-type difference-frequency (DF) and sum-frequency (SF) excitation and Raman-type Stokes and anti-Stokes probing. THz/NIR fields are color coded in magenta/green, respectively.

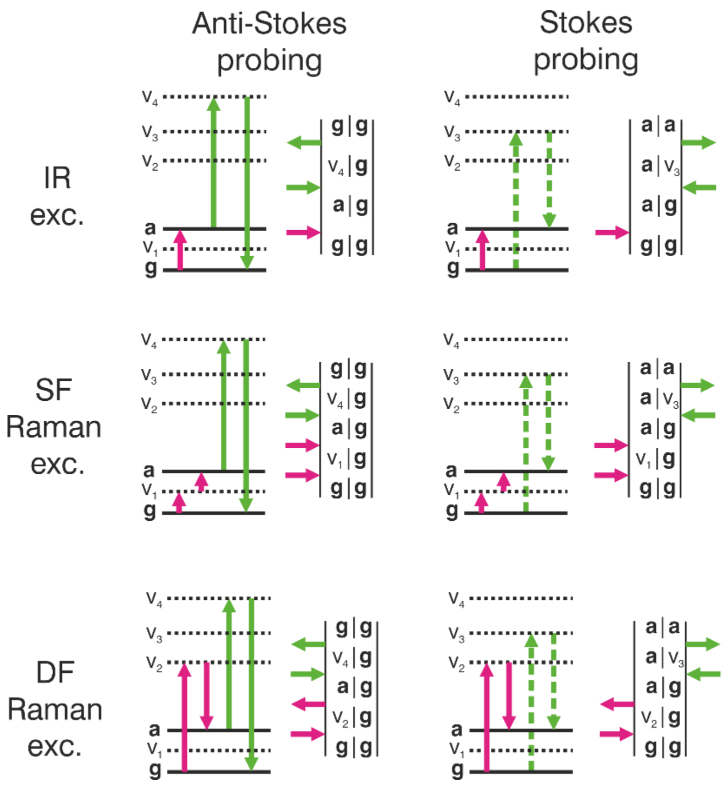

Figure S25.WMEL and Feynman diagrams for the nonlinear processes. v_1 … v_4 denote the virtual states, and **g** and **a** the ground and excited vibrational state, respectively.

References

[1] A. H. Proppe, G. W. Walters, A. Y. Alsalloum, A. A. Zhumekenov, E. Mosconi, S. O. Kelley, F. De Angelis, L. Adamska, P. Umari, O. M. Bakr, E. H. Sargent, *J. Phys. Chem. Lett.* **2020**, *11*, 716.

[2] F. Lédée, G. Trippé-Allard, H. Diab, P. Audebert, D. Garrot, J. S. Lauret, E. Deleporte, *CrystEngComm* **2017**, *19*, 2598.

[3] M. Frenzel, M. Cherasse, J. M. Urban, F. Wang, B. Xiang, L. Nest, L. Huber, L. Perfetti, M. Wolf, T. Kampfrath, X. Y. Zhu, S. F. Maehrlein, *Sci. Adv.* **2023**, *9*, eadg3856.

[4] S. F. Maehrlein, P. P. Joshi, L. Huber, F. Wang, M. Cherasse, Y. Liu, D. M. Juraschek, E. Mosconi, D. Meggiolaro, F. De Angelis, X. Y. Zhu, *Proc. Natl. Acad. Sci. U. S. A.* **2021**, *118*, e2022268118.

[5] L. Huber, S. F. Maehrlein, F. Wang, Y. Liu, X. Y. Zhu, *J. Chem. Phys.* **2021**, *154*, 094202.

[6] S. G. Motti, M. Kober-Czerny, M. Righetto, P. Holzhey, J. Smith, H. Kraus, H. J. Snaith, M. B. Johnston, L. M. Herz, *Adv Funct Mater.* **2023**, *33*, 2300363.

[7] X. Chen, H. Lu, Z. Li, Y. Zhai, P. F. Ndione, J. J. Berry, K. Zhu, Y. Yang, M. C. Beard, *ACS Energy Lett.* **2018**, *3*, 2273.

[8] M. Frenzel, J. M. Urban, L. Nest, T. Kampfrath, M. S. Spencer, S. F. Maehrlein, *Optica* **2024**, *11*, 362.

[9] M. Sajadi, M. Wolf, T. Kampfrath, *Opt. Express* **2015**, *23*, 28985.

[10] A. A. Lanin, I. V. Fedotov, A. B. Fedotov, D. A. Sidorov-Biryukov, A. M. Zheltikov, *Sci. Rep.* **2013**, *3*, 1842.

[11] T. Kohmoto, M. Masui, M. Abe, T. Moriyasu, K. Tanaka, *Phys. Rev. B - Condens. Matter Mater. Phys.* **2011**, *83*, 064304.

[12] S. Grisard, A. V. Trifonov, I. A. Solovev, D. R. Yakovlev, O. Hordiichuk, M. V. Kovalenko, M. Bayer, I. A. Akimov, *Nano Lett.* **2023**, *23*, 7397.

[13] M. Hase, K. Mizoguchi, H. Harima, S. Nakashima, K. Sakai, *Phys. Rev. B - Condens. Matter Mater. Phys.* **1998**, *58*, 5448.

[14] M. Balkanski, R. F. Wallis, E. Haro, *Phys. Rev. B* **1983**, *28*, 1928.

[15] P. G. Klemens, *Phys. Rev. B* **1975**, *11*, 3206.

[16] A. Cuquejo-Cid, A. García-Fernández, C. Popescu, J. M. Bermúdez-García, M. A. Señarís-Rodríguez, S. Castro-García, D. Vázquez-García, M. Sánchez-Andújar,

iScience **2022**, *25*, 104450.

[17] M. Hase, K. Ishioka, M. Kitajima, K. Ushida, S. Hishita, *Appl. Phys. Lett.* **2000**, *76*, 1258.

[18] R. Cuscó, E. Alarcón-Lladó, J. Ibáñez, L. Artús, J. Jiménez, B. Wang, M. J. Callahan, *Phys. Rev. B - Condens. Matter Mater. Phys.* **2007**, *75*, 165202.

[19] B. K. Ridley, *J. Phys. Condens. Matter* **1996**, *8*, 8.

[20] Z. Zhang, J. Zhang, Z.-J. Liu, N. S. Dahodl, W. Paritmongkol, N. Brown, Y.-C. Chien, Z. Dai, K. A. Nelson, W. A. Tisdale, A. M. Rappe, E. Baldini, *Sci. Adv.* **2023**, *9*, eadg4417.

[21] P. Ghosez, J. Michenaud, X. Gonze, *Phys. Rev. B - Condens. Matter Mater. Phys.* **1998**, *58*, 6224.

[22] D. M. Juraschek, S. F. Maehrlein, *Phys. Rev. B* **2018**, *97*, 174302.

[23] G. Khalsa, N. A. Benedek, J. Moses, *Phys. Rev. X* **2021**, *11*, 21067.

[24] S. Maehrlein, A. Paarmann, M. Wolf, T. Kampfrath, *Phys. Rev. Lett.* **2017**, *119*, 127402.

[25] D. M. Juraschek, D. S. Wang, P. Narang, *Phys. Rev. B* **2021**, *103*, 174302.

[26] T. E. Stevens, J. Kuhl, R. Merlin, *Phys. Rev. B - Condens. Matter Mater. Phys.* **2002**, *65*, 144304.

[27] M. Cho, *Phys. Rev. A - At. Mol. Opt. Phys.* **2000**, *61*, 12.

[28] H. Rigneault, P. Berto, *APL Photonics* **2018**, *3*, 091101.

[29] R. A. Bartels, D. Oron, H. Rigneault, *JPhys Photonics* **2021**, *3*, 042004.

[30] M. Frenzel, M. Cherasse, J. M. Urban, F. Wang, B. Xiang, L. Nest, L. Huber, L. Perfetti, M. Wolf, T. Kampfrath, X. Y. Zhu, S. F. Maehrlein, *Sci. Adv.* **2023**, *9*, eadg3856.

[31] R. Boyd, *Nonlinear Optics*, 3rd ed., Academic Press, Burlington, MA, USA, **2008**.

[32] F. Lédée, Cristallisation et fonctionnalisation de pérovskites hybrides halogénées à 2-dimensions pour le photovoltaïque et l'émission de lumière, Université Paris Saclay (COmUE). ⟨NNT : 2018SACLN045⟩. ⟨tel-01960754⟩, **2018**.

[33] D. J. Morrow, M. P. Hautzinger, D. P. Lafayette, J. M. Scheeler, L. Dang, M. Leng, D. D. Kohler, A. M. Wheaton, Y. Fu, I. A. Guzei, J. Tang, S. Jin, J. C. Wright, *J. Phys. Chem. Lett.* **2020**, *11*, 6551.

[34] A. P. Fellows, B. John, M. Wolf, M. Thämer, *Nat. Commun.* **2024**, *15*, 1.

[35] T. Khan, B. John, R. Niemann, A. Paarmann, M. Wolf, M. Thämer, *Opt. Express* **2023**, *31*, 28792.
